# Alpha-1 antitrypsin inhibits pertussis toxin

**DOI:** 10.1016/j.jbc.2024.107950

**Published:** 2024-10-30

**Authors:** Stefanie Lietz, Anja Sommer, Lena-Marie Sokolowski, Carolin Kling, Armando A. Rodríguez Alfonso, Nico Preising, Daniel Alpízar-Pedraza, Jaylyn King, Lisa Streit, Bernd Schröppel, Rene van Erp, Eberhard Barth, Marion Schneider, Jan Münch, Jens Michaelis, Ludger Ständker, Sebastian Wiese, Holger Barth, Arto T. Pulliainen, Karen Scanlon, Katharina Ernst

**Affiliations:** 1 Institute of Experimental and Clinical Pharmacology, Toxicology and Pharmacology of Natural Products, Ulm University Medical Center, Ulm, Germany; 2 Core Facility Functional Peptidomics, Faculty of Medicine, Ulm University, Ulm, Germany; 3 Core Unit Mass Spectrometry and Proteomics, Faculty of Medicine, Ulm University, Ulm, Germany; 4 Biochemistry and Molecular Biology Department, Center for Pharmaceutical Research and Development, Nuevo Vedado, Cuba; 5 Department of Microbiology and Immunology, University of Maryland School of Medicine, Baltimore, Maryland, USA; 6 Institute of Biophysics, Ulm University, Ulm, Germany; 7 Internal Medicine Clinic, Nephrology Section Core Facility, Ulm University Medical Center, Ulm, Germany; 8 Anesthesiology an Intensive Medicine Clinic, Ulm University Medical Center, Ulm, Germany; 9 Institute of Molecular Virology, Ulm University Medical Center, Ulm, Germany; 10 Institute of Biomedicine, University of Turku, Turku, Finland

**Keywords:** pertussis, whooping cough, infectious disease, *Bordetella pertussis*, bacterial toxin, pertussis toxin, α_1_-antitrypsin, inhibitor, toxin inhibitor, host defense, G-protein

## Abstract

Pertussis (whooping cough) is a vaccine-preventable but re-emerging, highly infectious respiratory disease caused by *Bordetella pertussis*. There are currently no effective treatments for pertussis, complicating care for nonvaccinated individuals, especially newborns. Disease manifestations are predominantly caused by pertussis toxin (PT), a pivotal virulence factor classified as an ADP-ribosylating AB-type protein toxin. In this work, an unbiased approach using peptide libraries, bioassay-guided fractionation and mass spectrometry revealed α_1_-antitrypsin (α_1_AT) as a potent PT inhibitor. Biochemistry-, cell culture-, and molecular modeling-based *in vitro* experimentation demonstrated that the α_1_AT mode of action is based on blocking PT-binding to the host target cell surface. In the infant mouse model of severe pertussis, α_1_AT expression was reduced upon infection. Further, systemic administration of α_1_AT significantly reduced *B. pertussis-*induced leukocytosis, which is a hallmark of infant infection and major risk factor for fatal pertussis. Taken together our data demonstrates that α_1_AT is a novel PT inhibitor and that further evaluation and development of α_1_AT as a therapeutic agent for pertussis is warranted. Importantly, purified α_1_AT is already in use clinically as an intravenous augmentation therapy for those with genetic α_1_AT deficiency and could be repurposed to clinical management of pertussis.

In 2014, the World Health Organization reported that pertussis, caused by *Bordetella pertussis*, affected more than 24.1 million children under five, resulting in 160,700 deaths globally (1). Despite the availability of vaccines and extensive vaccination coverage, especially in Western nations, pertussis is on the rise, with case numbers reaching unprecedented levels since the vaccine's introduction in the 1950s (1, 2). During the coronavirus disease 2019 pandemic, intervention measures enacted to mitigate the spread of Coronavirus disease 2019 had secondary effects on other infectious diseases, such as pertussis. However, latest data from 2023 for example from England indicate a rise of pertussis case numbers almost reaching prepandemic levels and are expected to rise in 2024 (https://www.gov.uk/government/publications/health-protection-report-volume-18-2024/hpr-volume-18-issue-1-news-1-february-2024). Moreover, Denmark is currently experiencing a whooping cough epidemic with substantial increase in case numbers in 2023. The European Centre for Disease Prevention and Control reports a significant increase in pertussis cases in the EU/EEA, with over 25,000 cases in 2023 and more than 32,000 between January and March 2024. Infants are the most affected group, with the highest incidence and mortality rates. Importantly, this infection burden significantly exceeds the levels observed during preceding recent epidemics (3, 4, https://www.ecdc.europa.eu/en/publications-data/increase-pertussis-cases-eueea). Hence, there is an urgent need for treatments to mitigate the severity of *B. pertussis-*induced disease.

Antibiotics, although effective in eradicating *B. pertussis* bacteria, do not alleviate the symptoms of pertussis unless administered within the first two weeks following infection. Such early treatment is rare due to delayed diagnoses (5). This is because toxins produced by *B. pertussis* rather than the bacterium itself cause the majority of disease manifestations. Pertussis toxin (PT) plays a vital role as a virulence factor in causing whooping cough, a disease marked by a long-lasting (around ten weeks) and uncontrollable cough (6, 7). This illness can lead to secondary problems like vomiting and pneumothorax, and in more severe instances, may cause leukocytosis, pneumonia, encephalopathy, seizures, or apnea. These serious conditions can be life-threatening, especially for newborns and young infants (5, 8).

Research has shown that PT is responsible for extended and severe inflammation in the airways, for example, in mouse models (9, 10). Strains of *B. pertussis* lacking PT expression did not result in severe symptoms like leukocytosis or death, underscoring PT's critical role, particularly in severe cases (6). Consequently, PT is considered a promising focus for creating innovative therapeutic approaches.

The AB_5_ protein toxin produced by *B. pertussis*, known as PT, is made up of an enzymatic component, the A-protomer PTS1, and a five-part B-subunit (11, 12, 13). This B-subunit is formed by PTS2, PTS3, PTS4, and PTS5 in a ratio of 1:1:2:1. PTS1 associates with the B pentamer *via* noncovalent bonds in the periplasm of *B. pertussis* (11). The components together create a PT holotoxin with a pyramid-like structure that is secreted by the bacteria through a type-IV secretion system (11, 14). The B pentamer allows the binding of PT to sialoglycoproteins on target cell surfaces, facilitating entry through receptor-mediated endocytosis (15, 16). PT then follows a retrograde intracellular transport route from the Golgi apparatus to the endoplasmic reticulum (ER) (17, 18). In the ER, ATP attaches to the central pore of the B pentamer, inducing a change in its structure, which subsequently triggers the release of PTS1 (19, 20, 21). In its detached form, PTS1 is thermally unstable and transitions to a disordered state (22, 23). This unfolded PTS1 is then identified as an ER-associated degradation substrate and transported from the ER into the cytosol. Lacking lysine residues, PTS1 is safeguarded from ubiquitin-dependent degradation by the proteasome (22, 23, 24). The transport of PTS1 into the host cell cytosol relies on the assistance of host cell chaperones and enzymes that aid in the folding of proteins (25, 26, 27, 28). In the cytosol, PTS1 transfers an ADP-ribose moiety from NAD^+^ onto the α-subunit of inhibitory G proteins (Gαi), resulting in Gαi's inability to connect with G-protein-coupled receptors. This process causes, for instance, a heightened cAMP level due to the lack of Gαi-mediated inhibition of adenylate cyclase activity (29, 30).

The lack of treatment options for whooping cough necessitates the exploration of novel inhibitors against PT. Here, we searched for novel PT-inhibitors in the human peptidome. The human peptidome refers to the complete collection of peptides in the human body. Endogenous peptides and small proteins have various functions. They play a role as a first line of defense against invading microbes (*e.g.*, antimicrobial peptides), regulate the immune system, or have anticancer properties. The inherent potential of these peptides for drug development lies in their innate evolutionary optimization, specifically tailored to carry out their respective functions effectively in the human body (31). To identify PT-inhibitory peptides/proteins, we generated peptide/protein library derived from human hemofiltrate and screened it for inhibitors of PT function. This allowed us to discover α_1_-antitrypsin (α_1_AT), a highly abundant serine protease inhibitor, as a novel PT inhibitor. α_1_AT is a crucial protein produced by the liver that plays a vital role in protecting the lungs from damage caused by enzymes like neutrophil elastase. α_1_AT deficiency is a genetic disorder that leads to unregulated tissue degradation in the lower respiratory tract and requires intravenous replacement therapy. Therefore, α_1_AT purified from donor blood (*e.g.*, Prolastin) is used as a licensed drug to treat this deficiency (32). This finding is suggestive of an opportunity to repurpose α_1_AT-containing drugs potentially gaining a new indication for treating pertussis.

## Results

### α_1_AT was identified from a hemofiltrate library as a PT inhibitor

**Figure 1:**
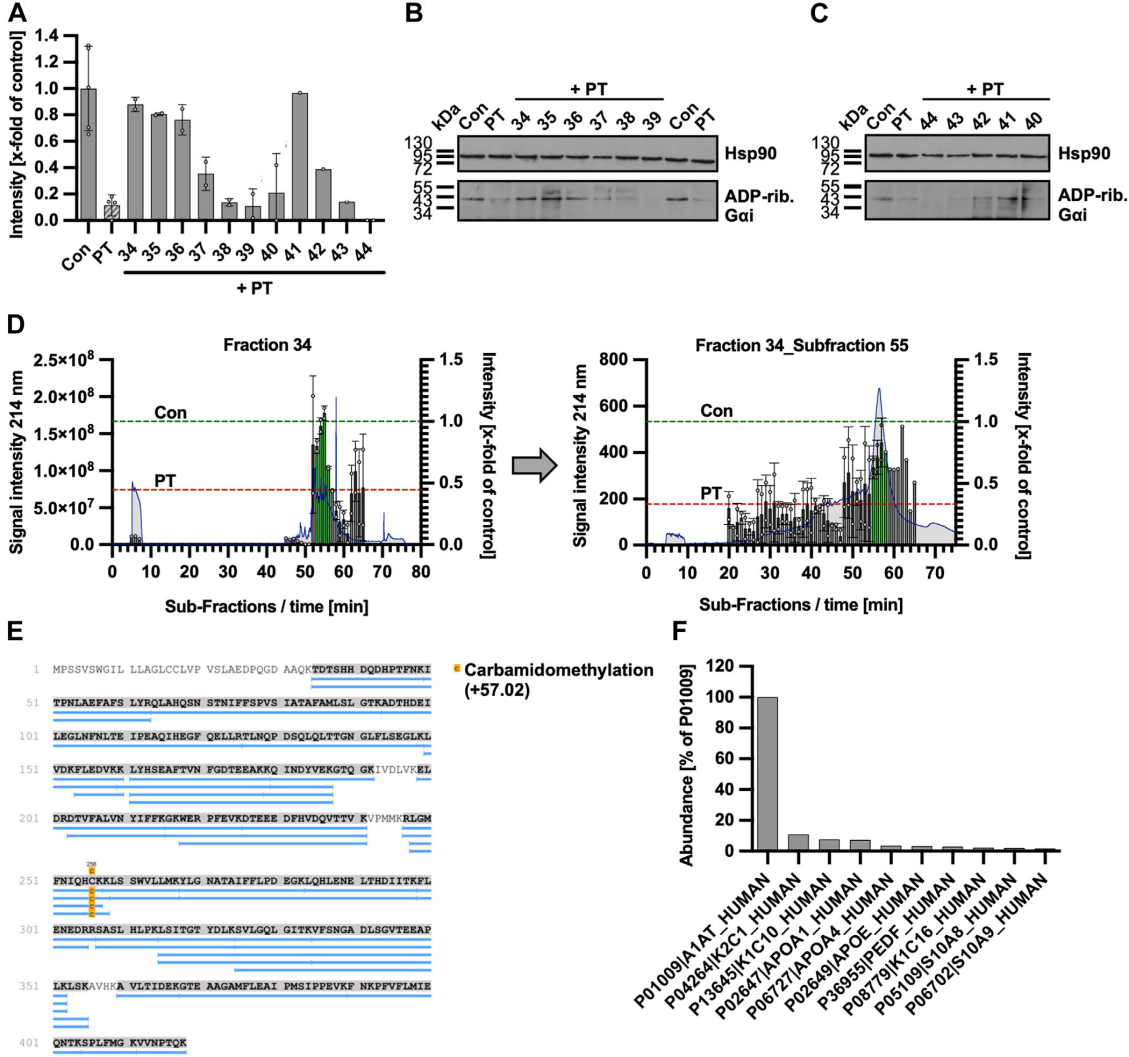
**Alpha-1-antitrypsin (α**_**1**_**AT) was identified from hemofiltrate library as PT inhibitor.***A*–*C*, PT (10 ng/ml = 0.0000962 μM) and 20 μl of a fraction of the hemofiltrate library or the respective amount of solvent (H_2_O) were added directly to CHO-K1 cells in FCS-free medium and incubated for 4 h at 37 °C. Cells were left untreated as further control. Then, the cells were lysed and Gαi which has not been ADP-ribosylated during the intoxication with PT was ADP-ribosylated and biotin-labeled *via* the incubation with PTS1 and biotin-labeled NAD^+^. Subsequently, the biotin-labeled Gαi was detected *via* Western Blot, while Hsp90 served as control for equal protein loading. The bar graph (*A*) shows the quantifications of Western Blot signals from one experiment testing the samples as single values or duplicates depending on sample availability, while (*B*, *C*) show the corresponding Western blot images. The intensity values of the bar graph are given as x-fold of the untreated control (Con), normalized to Hsp90, mean ± SD (n ≥ 1). *D*, PT (10 ng/ml) and 20 μl of the respective chromatographic subfractions or the respective amount of solvent (H_2_O) were added directly to CHO-K1 cells in FCS-free medium and incubated for 4 h at 37 °C. Cells were left untreated as further control. Subsequently, the experiment was performed as described in (*A*). The intensity values of the bar graph are given as x-fold of the untreated control (Con), normalized to Hsp90 staining, mean ± SD (at least n = 1 at most n = 2 from two independent experiments). (*Blue line*: chromatogram from the chromatographic fractionation process, *gray bars* = screening result, *green bars*: screening results that were considered as hits and or subjected to further chromatographic fractionation). *E*, sample carbamidomethylation, followed by proteolytic digestion, and mass spectrometry analysis revealed the presence of α_1_AT. The sequence coverage compared to the mature polypeptide was 93.5% with a α_1_AT precursor amino acid sequence range of 25 to 418. *F*, α_1_AT (Uniprot: P01009-A1AT_HUMAN) was the main component of the active chromatographic subfractions 34_55_56 to 58. Abbreviations are used to identify all chromatographic fractions. From *left* to *right*, the number of the active chromatographic fraction used for each next fractionation step is given in chronological order. Therefore, 34_55_56 to 58 is referred to as the chromatographic fractions 56 to 58 derived from fraction 34_55, obtained from fraction 34. Subfractions 34_55_56 to 58 are 56, 57, and 58, obtained from 34_55 (Fig. 1*C*, *right* side), which showed the highest activity and were subjected together to sequencing. We used this nomenclature to abbreviate the names. The same applies to the other names, for example, 35_48 to 49 indicates fractions 48 and 49 from the fractionation of 35. FCS, fetal calf serum; Gαi, α-subunit of inhibitory G protein.

A peptide/protein library derived from human hemofiltrate was screened for inhibitors against PT. The initial library was generated by a serial combination of cation-exchange chromatography and reversed-phase chromatography from 1000 l human hemofiltrate and consisted of 440 fractions containing a multitude of peptides/proteins (Fig. S1). To assess the effect of the different library fractions on the toxicity of PT, a cell-based assay was used where the ADP-ribosylation status of the Gαi substrate protein of PT was analyzed. Therefore, Chinese hamster ovary cells strain K1 (CHO-K1) cells were incubated with PT and the respective library fractions on cells for 4 h. Cell lysates were generated and incubated with recombinant PTS1 and biotin-labeled cosubstrate NAD^+^. This allowed for ADP-ribosylation and thereby biotin-labeling of Gαi that was not previously modified in the living cells. Biotin-labeled Gαi was detected by Western blotting. Untreated control samples showed a strong signal because no Gαi had been ADP-ribosylated in the PT-treated living cells and therefore served as a substrate in the subsequent *in vitro* ADP-ribosylation assay (Fig. 1, *A*–*C*). Samples treated with PT revealed a significantly weaker signal. Fig. 1, *A*–*C* shows the hit fractions 34, 35, 36, and 41 (from the fractionation of CEX pool E4), identified in the first round of screening of the original peptide/protein bank. Results of other tested fractions of the original peptide/protein bank are not shown. The original hit fractions 34, 35, 36, and 41 were then subjected to further chromatographic fractionation, resulting in novel subfractions that were screened again (Fig. S2, *A*–*D*). Subfractions of the original fractions 34, 35, and 36 were positive for PT inhibition during screening and were accordingly subjected to further chromatographic fractionation resulting in subsubfractions (Fig. S2, *C*–*J*). After two rounds of bioassay-guided fractionations (Fig. 1*D*), mass spectrometry sequencing analysis allowed the identification of α_1_AT (Fig. 1, *E* and *F*), a member of the serine protease inhibitor (serpin) super-family as the dominant protein in active chromatographic subfractions 34_55_56 to 58 (= fraction 34_subfraction 55_subsubfraction 56–58).

### α_1_AT inhibits intoxication of CHO-K1 and A549 cells with PT in a concentration-dependent manner

In the following experiments, we used the plasma-derived α_1_AT, available as approved drug (Prolastin), to unravel the underlying mechanism of PT inhibition.

**Figure 2:**
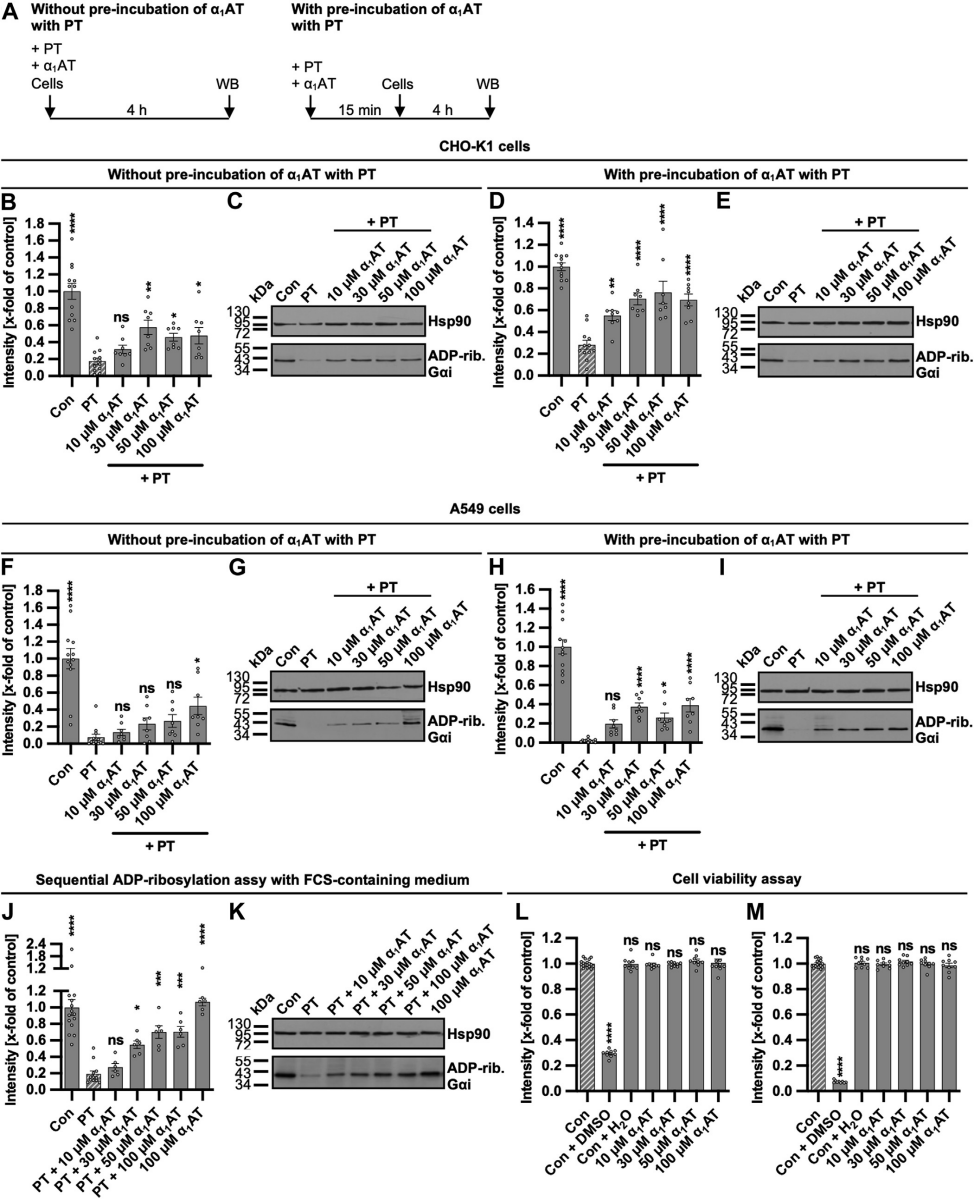
**Effect of α**_**1**_**AT on ADP-ribosylation status of Gαi in PT-treated CHO-K1 and A549 cells.***A*, schematic representation of experimental setup for the sequential ADP-ribosylation assay. PT and α_1_AT were either directly added to cells for 4 h or preincubated for 15 min before addition to cells. *B*–*C*, PT (10 ng/ml) and different concentrations of α_1_AT or the respective amount of solvent (H_2_O) were added directly to CHO-K1 cells in FCS-free medium and incubated for 4 h at 37 °C. Cells were left untreated as further control. After the incubation, the cells were lysed and Gαi, which had not been ADP-ribosylated during the intoxication with PT, was ADP-ribosylated and biotin-labeled *via* the incubation with PTS1 and biotin-labeled NAD^+^. Subsequently, the biotin-labeled Gαi was detected *via* Western blot, while Hsp90 served as a control for equal protein loading. The bar graph (*B*) shows the quantification of Western blot signals from four independent experiments, while (*C*) shows results of a representative experiment. The intensity values of the bar graph are given as x-fold of the untreated control (Con), normalized to Hsp90, mean ± SEM (n = 8 values from four independent experiments). *D* and *E*, PT (10 ng/ml) and different concentrations of α_1_AT or the respective amount of solvent (H_2_O) were preincubated for 15 min at room temperature and added to CHO-K1 cells in FCS-free medium for 4 h at 37 °C. Cells were left untreated as further control. Subsequently, the experiment was performed as described in (*B* and *C*). Values are given as mean ± SEM (n = 8 values from four independent experiments). *F* and *G*, PT (50 ng/ml = 0.000476 μM) and different concentrations of α_1_AT or the respective amount of solvent (H_2_O) were added directly to A549 cells in FCS-free medium and incubated for 4 h at 37 °C. Cells were *left* untreated as further control. Subsequently, the experiment was performed as described in (*B* and *C*). Values are given as mean ± SEM (n = 8 values from four independent experiments). *H* and *I*, PT (50 ng/ml) and different concentrations of α_1_AT or the respective amount of solvent (H_2_O) were preincubated for 15 min at room temperature and added to A549 cells in FCS-free medium for 4 h at 37 °C. Cells were *left* untreated as further control. Subsequently, the experiment was performed as described in (*B* and *C*). Values are given as mean ± SEM (n = 8 values from four independent experiments). *J* and *K*, PT (10 ng/ml) and different concentrations of α_1_AT or the respective amount of solvent (H_2_O) were added directly to CHO-K1 cells in FCS-containing medium and incubated for 4 h at 37 °C. Cells were *left* untreated as further control. Subsequently, the experiment was performed as described in (*B* and *C*). Values are given as mean ± SEM (n = 6 values from three independent experiments). *L* and *M*, different concentrations of α_1_AT or the respective amount of solvent (H_2_O) were added directly to CHO-K1 cells in FCS-free medium and incubated for 4 h (*L*) or 24 h (*M*) at 37 °C. As a positive control for cell death, 20% DMSO was added. After the incubation, the MTS reagent was added to the cells and incubated for 45 min at 37 °C. The absorbance was measured using a plate reader at 490 nm. The intensity values of the bar graph are given as x-fold of the untreated control (Con), mean ± SEM (n = 9 values from three independent experiments). (*B*, *D*, *F*, *H*, *J*, *L*, and *M*) significance was tested using one-way ANOVA followed by Dunnett’s multiple comparison test and refers to samples treated with PT only (*B*, *D*, *F*, *H*, and *J*) or untreated control (Con) (*L* and *M*) (∗*p* < 0.1, ∗∗*p* < 0.01, ∗∗∗*p* < 0.001, ∗∗∗∗*p* < 0.0001, ns not significant). α_1_AT, α_1_-antitrypsin; DMSO, dimethylsulfoxide; FCS, fetal calf serum; Gαi, α-subunit of inhibitory G protein; MTS, 3-(4,5-dimethylthiazol-2-yl)-5-(3-carboxymethoxyphenyl)-2-(4-sulfophenyl)-2H-tetrazolium; PT, pertussis toxin; WB, Western blot.

First, the inhibiting effect of α_1_AT on the intoxication of CHO-K1 cells and lung adenoma A549 cells was confirmed by analyzing the ADP-ribosylation status of Gαi (Fig. 2). Inhibition was more pronounced if α_1_AT and PT were preincubated for 15 min compared to the simultaneous addition of both components to the cells. A concentration-dependent inhibition was observed under most conditions. Experiments have been conducted in a serum-free medium to efficiently detect inhibitory peptides/proteins, ensuring they are not missed due to interactions with serum proteins. However, the inhibiting and concentration-dependent effect of α_1_AT on PT-intoxication of cells was also observed in the presence of serum (Fig. 2, *J* and *K*). α_1_AT had no effect on the cell viability of CHO-K1 cells after 4 or 24 h of incubation (Fig. 2, *L* and *M*). Moreover, treatment of cells with only α_1_AT had no impact on the subsequent detection of ADP-ribosylated Gαi (Fig. S3).

**Figure 3:**
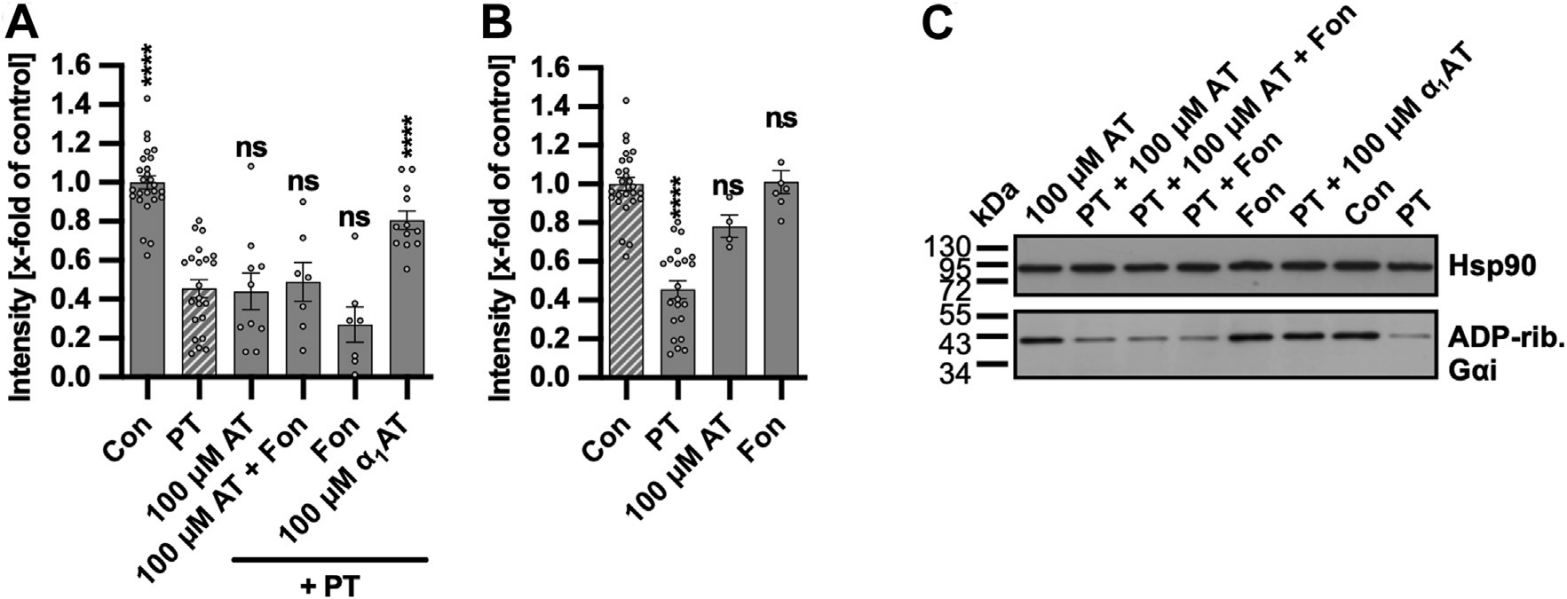
**Effect of the serpin proteins α**_**1**_**AT and antithrombin on intoxication of CHO-K1 cells with PT.***A*–*C*, PT (10 ng/ml) and α_1_AT (100 μM), antithrombin (AT) (100 μM), and fondaparinux (Fon) (1 g/l) or the respective amount of solvent (H_2_O) were added directly to CHO-K1 cells in FCS-free medium and incubated for 4 h at 37 °C. Cells were left untreated as further control (Con) or incubated with AT and Fon only. After the incubation, the cells were lysed and Gαi which has not been ADP-ribosylated during the intoxication with PT was ADP-ribosylated and biotin-labeled *via* the incubation with PTS1 and biotin-labeled NAD^+^. Subsequently, the biotin-labeled Gαi was detected *via* Western Blot, while Hsp90 or Ponceau-S staining served as control for equal protein loading. The bar graphs (*A* and *B*) show the quantifications of Western blot signals from six independent experiments, while (*C*) shows blots of a representative experiment. The intensity values of the bar graph are given as x-fold of the untreated control (Con), normalized to Hsp90 or Ponceau-S staining, mean ± SEM (at least n = 7 values from six independent experiments). *A* and *B*, significance was tested using one-way ANOVA followed by Dunnett’s multiple comparison test and refers to controls treated with PT only (*A*) or untreated controls (Con) (*B*) (∗*p* < 0.1, ∗∗*p* < 0.01, ∗∗∗*p* < 0.001, ∗∗∗∗*p* < 0.0001, ns not significant). α_1_AT, α_1_-antitrypsin; Gαi, α-subunit of inhibitory G protein; PT, pertussis toxin.

In the initial step toward understanding the inhibition mechanism, we investigated whether another member of the serpin superfamily (family of proteins with protease activity), antithrombin, possesses the ability to inhibit PT intoxication. This was performed to investigate whether the protease activity might be required for the mechanism of PT inhibition. Although α_1_AT and antithrombin are both members of the serpin superfamily they do not share the same physiological targets. α_1_AT physiological targets are the neutrophil elastase and trypsin. Antithrombin is a natural protein in the blood that plays a critical role in regulating blood clot formation by inhibiting the activity of various clotting factors, particularly thrombin and factor Xa (33). Notably, the drug fondaparinux binds to antithrombin, enhancing its inhibition of factor Xa by 300-fold (34). Neither antithrombin nor fondaparinux, alone or combined with antithrombin, showed any inhibitory effect on the PT intoxication of cells (Fig. 3). This observation suggests that the inhibition of PT may not be reliant on α_1_AT’s protease inhibitor activity since another protease, antithrombin, does not have inhibitory effects on PT.

### α_1_AT has a minor influence on the enzyme activity of PTS1

**Figure 4:**
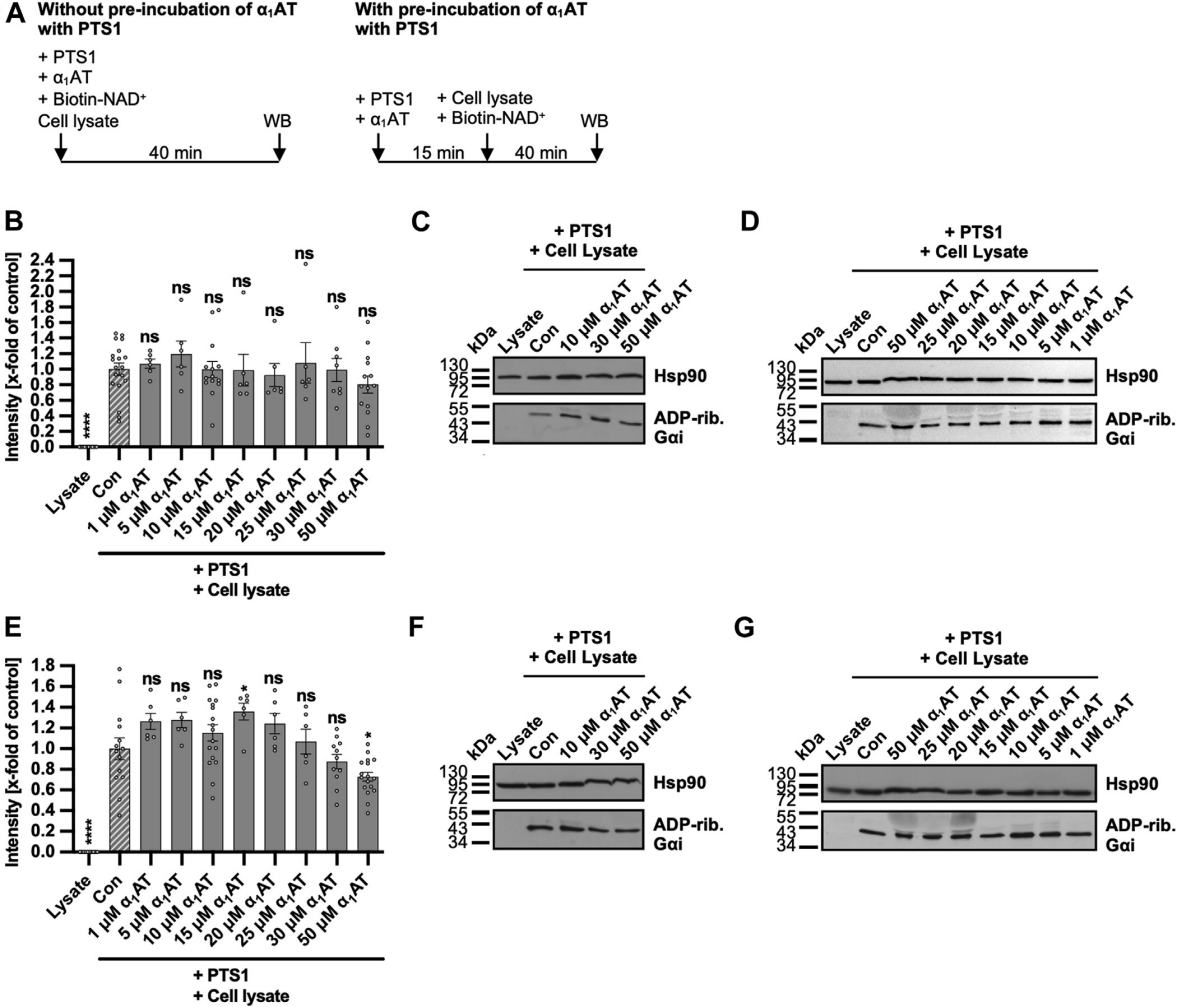
**Effect of α**_**1**_**AT on enzyme activity of PTS1 *in vitro*.***A*, schematic representation of experimental setup for the enzyme activity assay. PTS1 and α_1_AT were either directly added to CHO-K1 cell lysate with biotin-NAD^+^ and incubated for 40 min or preincubated for 15 min before addition to cell lysate and biotin-NAD^+^. *B*–*D*, PTS1 (84 nM) and different concentrations of α_1_AT or the respective amount of solvent (H_2_O) (Con) were added directly to CHO-K1 cell lysate and biotin-NAD^+^ and incubated for 40 min at room temperature. Cell lysate was left untreated with biotin-NAD^+^ as further control (lysate). Gαi, which was ADP-ribosylated and biotin-labeled *via* the incubation with PTS1 and biotin-labeled NAD^+^, was detected *via* Western blot, while Hsp90 or Ponceau-S staining served as control for equal protein loading. The bar graph (*B*) shows the quantifications of Western blot signals from six independent experiments, while (*C* and *D*) show blots of representative experiments. The intensity values of the bar graph are given as x-fold of the control (Con), normalized to Hsp90 or Ponceau-S staining, mean ± SEM (at least n = 6 values from six independent experiments). *E*–*G*, PTS1 (84 nM) and different concentrations of α_1_AT or the respective amount of solvent (H_2_O) (Con) were preincubated for 15 min at room temperature before addition to CHO-K1 cell lysate and biotin-NAD^+^. Cell lysate was left untreated with biotin-NAD^+^ as further control (lysate). Subsequently, the experiment was performed as described in (*B*–*D*). The bar graph (*E*) shows the quantifications of Western blot signals from seven independent experiments, while (*F* and *G*) show blots of representative experiments. The intensity values of the bar graph are given as x-fold of the control (Con), normalized to Hsp90 or Ponceau-S staining, mean ± SEM (at least n = 6 values from seven independent experiments). *B* and *E*, significance was tested using one-way ANOVA followed by Dunnett’s multiple comparison test and refers to controls treated with PT only (∗*p* < 0.1, ∗∗*p* < 0.01, ∗∗∗*p* < 0.001, ∗∗∗∗*p* < 0.0001, ns not significant). α_1_AT, α_1_-antitrypsin; Gαi, α-subunit of inhibitory G protein; PT, pertussis toxin; WB, Western Blot.

To assess the impact of α_1_AT on the enzyme activity of PTS1, which is essential for intoxication, PTS1 was incubated in cell lysate with or without varying concentrations of α_1_AT in the presence of biotin-labeled NAD^+^. The cell lysate served as a source of Gαi. ADP-ribosylated and thus biotin-labeled Gαi was detected by Western blotting (Fig. 4). Higher concentrations of α_1_AT (>50 μM) proved challenging to analyze in this assay, as they affected the protein migration in SDS-page (Fig. 4, *C*, *D*, *F* and *G*). Consequently, only concentrations up to 50 μM α_1_AT were included in this analysis, and these concentrations demonstrated only minor effects on PTS1 enzyme activity *in vitro*. Noteworthy, only the samples treated with 50 μM α_1_AT preincubated with PTS1 (Fig. 4*E*) displayed a slight reduction of signal. However, this minimal effect is unlikely to account for the substantial inhibition of cell intoxication by α_1_AT. Additionally, the enzyme activity was tested *in vitro* using recombinant Gαi also showing only minor effects (Fig. S4).

### α_1_AT inhibits binding of labeled PT to CHO-K1 and A549 cells in a concentration-dependent manner

**Figure 5:**
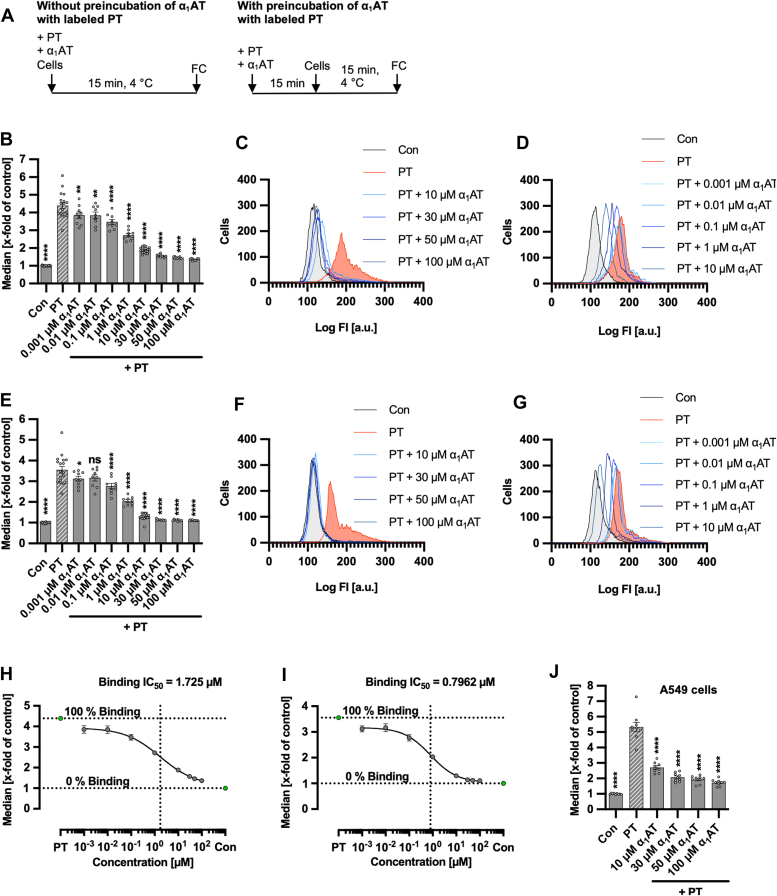
**Effect of α**_**1**_**AT on binding of labeled PT to CHO-K1 and A549 cells.***A*, schematic representation of experimental setup for the flow cytometry (FC) based binding assay. The 488-labeled PT and α_1_AT were either directly added to cells for 15 min or preincubated for 15 min before addition to cells. *B*–*D*, 488-labeled PT (500 ng/ml = 0.00476 μM) and different concentrations of α_1_AT or the respective amount of solvent (H_2_O) were added directly to CHO-K1 cells and incubated for 15 min at 4 °C to enable binding of PT to cells but prevent internalization. Cells were left untreated as control (Con). After that, cells were washed by centrifugation and 488-labeled PT bound to cell surfaces was measured using flow cytometry. Values of median are given as x-fold of the untreated control (Con), mean ± SEM (n = 9 values from three independent experiments). Histograms show fluorescence intensity of cells for one representative experiment for higher (*C*) and lower (*D*) concentrations (n = 3). *E*–*G*, The 488-labeled PT (500 ng/ml) and different concentrations of α_1_AT or the respective amount of solvent (H_2_O) were preincubated for 15 min at room temperature before addition to CHO-K1 cells. Cells were *left* untreated as control, and the experiment was performed as described in (*B*–*D*). Values of median are given as x-fold of the untreated control (con), mean ± SEM (n = 9 values from three independent experiments). Histograms show fluorescence intensity of cells for one representative experiment for higher (*F*) and lower (*G*) concentrations (n = 3). *H*–*I*, binding IC_50_ values were calculated from median fluorescence intensities for experiments without (*I*, *H*) and with (*E*, *I*) the preincubation of 488-labeled PT and different concentrations α_1_AT. A nonlinear regression model with variable slope (GraphPad Prism, log(inhibitor) *versus* response (variable slope, four parameters)) was fitted to values, and binding IC_50_ values were given based on the fit. *j*, 488-labeled PT (1000 ng/ml = 0.00962 μM) and different concentrations of α_1_AT or the respective amount of solvent (H_2_O) were added directly to A549 cells. Cells were left untreated as control (Con) and the experiment was performed as described in (b-d). Values of median are given as x-fold of the untreated control (Con), mean ± SEM (n = 9 values from three independent experiments). *B*, *E*, and *J*, significance was tested using one-way ANOVA followed by Dunnett’s multiple comparison test and refers to samples treated with PT only (*B*, *E*, and *J*) (∗*p* < 0.1, ∗∗*p* < 0.01, ∗∗∗*p* < 0.001, ∗∗∗∗*p* < 0.0001, ns not significant). α_1_AT, α_1_-antitrypsin; PT, pertussis toxin.

The effect of α_1_AT on binding of PT to cells was investigated by using fluorescence-labeled PT in flow cytometry analysis. Therefore, PT was added to cells in the presence of α_1_AT either after preincubation or directly. Cells were incubated at 4 °C during the experiment to allow binding but prevent internalization. After extensive washing of cells, bound PT was detected by flow cytometry, which revealed a significant, concentration-dependent inhibition of PT-binding to cells by α_1_AT, regardless of whether a preincubation was performed or not (Fig. 5). However, determination of IC_50_ values showed that when labeled PT was preincubated with α_1_AT, binding IC_50_ values were more than two-fold lower compared to when labeled PT and α_1_AT were added directly to cells (Fig. 5, *H* and *I*). This suggests that interaction of PT and α_1_AT during the preincubation might be relevant for better inhibition of PT. A significant inhibition of PT-binding by α_1_AT was also observed in A549 cells (Fig. 5*J*).

**Figure 6:**
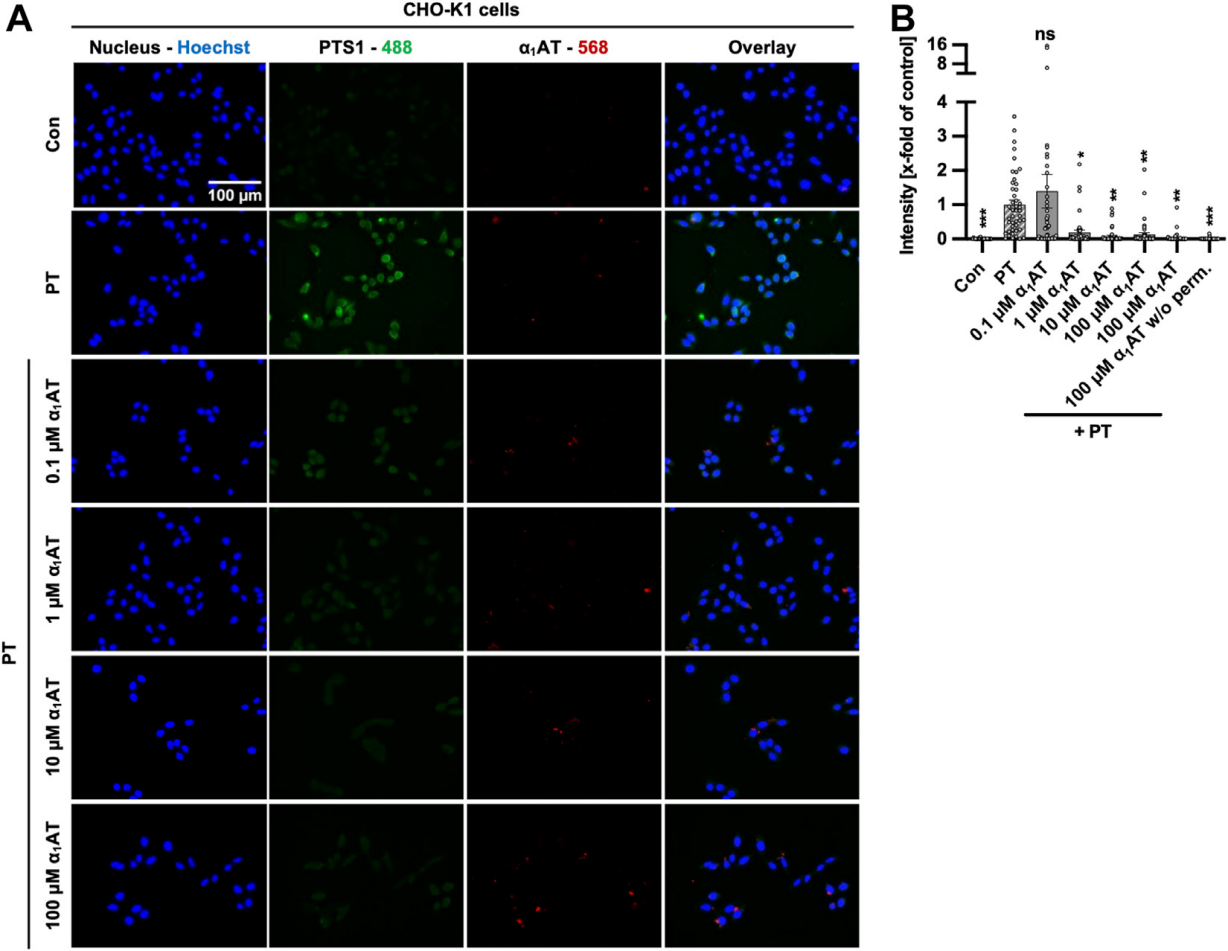
**Effect of α**_**1**_**AT on binding of PT in CHO-K1 cells.***A*, PT (1 μg/ml = 0.00952 μM) and different concentrations of α_1_AT or the respective amount of solvent (H_2_O) were added directly to CHO-K1 cells and incubated for 40 min at 4 °C to enable PT binding but not internalization. Cells were left untreated as control. Subsequently, the cells were washed, fixed, permeabilized, and quenching was performed. Blocking was performed and the cells were incubated with primary antibodies for PTS1 (*green*) and α_1_AT (*red*). Primary antibodies were detected *via* fluorescently labeled secondary antibodies and nuclei were stained using Hoechst (*blue*). Representative images are shown from three independent experiments (n = 3), (*B*) whereas the PTS1 signal is given in the bar graph as x-fold of the PT-treated control (PT), normalized to the Hoechst signal, mean ± SEM (n = 44–45 values from three independent experiments). Significance was tested using one-way ANOVA followed by Dunnett’s multiple comparison test and refers to samples treated with PT only (∗*p* < 0.1, ∗∗*p* < 0.01, ∗∗∗*p* < 0.001, ∗∗∗∗*p* < 0.0001, ns not significant). α_1_AT, α_1_-antitrypsin; PT, pertussis toxin.

Inhibition of PT-binding to cells was also confirmed by detection of PTS1 *via* immunofluorescence microscopy in cells that were incubated at 4 °C with PT in the presence or absence of α_1_AT (Fig. 6). Here too, a reduction of PTS1 signal was observed with increasing concentrations of α_1_AT, showing that binding of PT to cells is impaired by α_1_AT.

### α_1_AT reduces detectable PTS1 signal in CHO-K1 and A549 cells in a concentration-dependent manner

**Figure 7:**
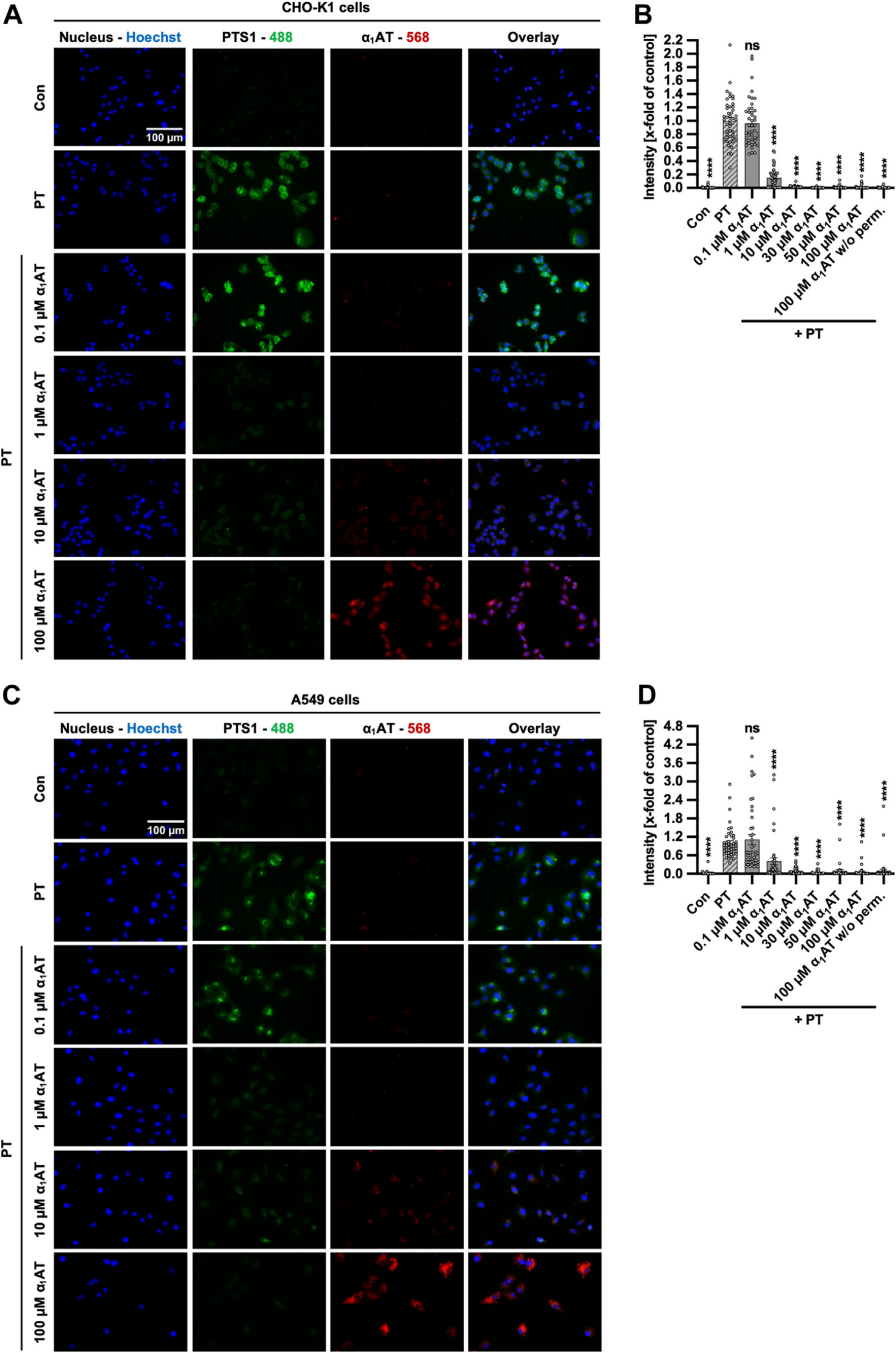
**Effect of α**_**1**_**AT on detectable PTS1 signal in CHO-K1 and A549 cells.***A*–*B*, PT (100 ng/ml = 0.000952 μM) and different concentrations of α_1_AT or the respective amount of solvent (H_2_O) were added directly to CHO-K1 cells and incubated for 4 h at 37 °C. Cells were left untreated as control (Con). Subsequently, the cells were washed, fixed, permeabilized, and quenching was performed. After blocking, cells were incubated with primary antibodies against PTS1 (*green*) and α_1_AT (*red*). Primary antibodies were detected *via* fluorescently labeled secondary antibodies, and nuclei were stained using Hoechst (*blue*). Representative images are shown from three independent experiments (n = 3), *B*, PTS1 signal is given in the bar graph as x-fold of the PT-treated control (PT), normalized to the Hoechst signal, mean ± SEM (n = 45 values from three independent experiments). *C*, PT (100 ng/ml) and α_1_AT or the respective amount of solvent (H_2_O) were added directly to A549 cells and incubated for 4 h at 37 °C. Subsequently, the experiment was performed as described in (*A*). Representative images are shown from three independent experiments (n = 3), *D*, PTS1 signal is given in the bar graph as x-fold of the PT-treated control (PT), normalized to the Hoechst signal, mean ± SEM (n = 43–45 values from three independent experiments). *B* and *D*, significance was tested using one-way ANOVA followed by Dunnett’s multiple comparison test and refers to samples treated with PT only (∗*p* < 0.1, ∗∗*p* < 0.01, ∗∗∗*p* < 0.001, ∗∗∗∗*p* < 0.0001, ns not significant). α_1_AT, α_1_-antitrypsin; PT, pertussis toxin.

For the intoxication of cells by PT, it is crucial that PTS1 is taken up into cells and reaches its specific substrate Gαi in the cytosol. Therefore, PTS1 detection was analyzed in cells incubated at 37 °C, thus allowing cellular uptake of the toxin. Fig. 7 shows that, in the presence of α_1_AT, the signal for PTS1 is strongly decreased in CHO-K1 cells and in A549 cells. Additional concentrations of 30 and 50 μM α_1_AT were tested, and they also demonstrated a decreased signal of PTS1 in both cell lines (Fig. S5). Furthermore, cells were incubated with 100 μM α_1_AT without PT. After fixation, we followed the same staining protocol as for the previous samples, with the exception that cells were not permeabilized, using 0.4% (v/v) Triton X-100 in PBS for 5 min. The fluorescence signal obtained for α_1_AT was comparable to samples that had been permeabilized, suggesting that α_1_AT is not internalized by the cells but rather binds to the cell surface (Fig. S5). If α_1_AT would be taken up and be located within the cells, no signal for α_1_AT would be expected, since by skipping the permeabilization process, antibodies were not able to reach the cytosol. The same procedure was performed at 4 °C to allow binding but not internalization, and again, the signal intensity of α_1_AT was comparable, independent of permeabilization (Fig. S6). Moreover, high-resolution stimulated emission depletion (STED) microscopy was performed, which confirmed that the PTS1 signal decreases in CHO-K1 cells in the presence of α_1_AT (Fig. S7). Additionally, the PTS1 and α_1_AT signals indicate a colocalization, especially prominent using higher concentrations of α_1_AT (Fig. S7, indicated by arrows).

### Cell-bound PT displays limited displacement and no inhibition from uptake by α_1_AT

**Figure 8:**
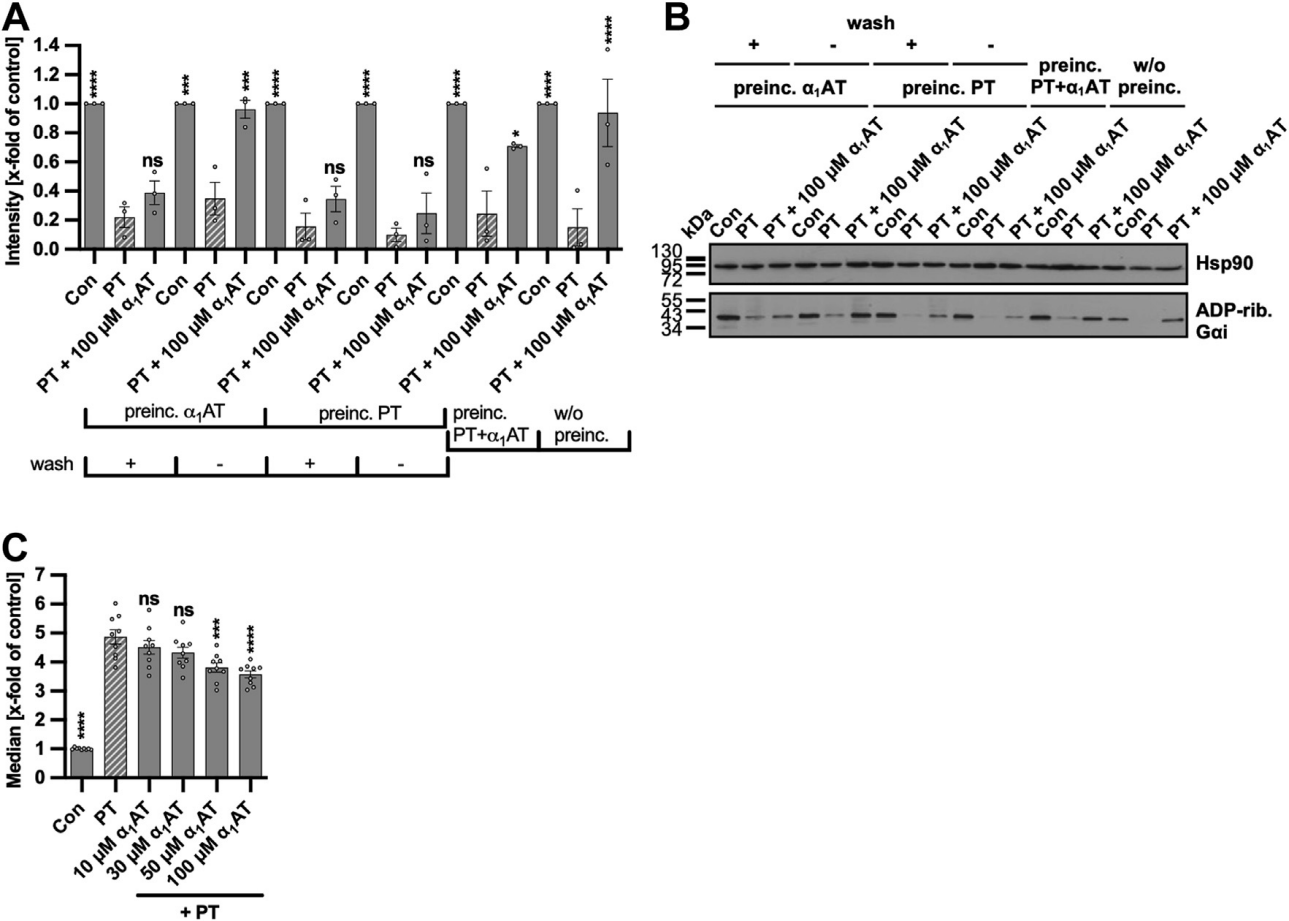
**Effect of different preincubation schemes of α**_**1**_**AT on PT-intoxication and binding of PT to cells.***A*–*B*, for the competition study based on the sequential ADP-ribosylation of Gαi six different treatment options using PT (10 ng/ml) and α_1_AT (100 μM) or the respective amount of solvent (H_2_O) were performed in parallel. First and second, α_1_AT or solvent control were preincubated on CHO-K1 cells for 15 min at 37 °C and were either present in the medium during intoxication with PT or washed away prior to PT-intoxication. Third and fourth, PT was preincubated on cells for 15 min at 37 °C and was either present in the medium while α_1_AT or solvent control were added or washed away before adding α_1_AT or water. Fifth, α_1_AT, water (solvent control), and PT were preincubated for 15 min at room temperature before addition to CHO-K1 cells. Sixth, α_1_AT, solvent control, and PT were added directly to CHO-K1 cells. Cells were *left* untreated as further control (Con). The wash steps were performed using FCS-free medium. After the CHO-K1 cells were treated, the cells were incubated for further 4 h at 37 °C. Then, the cells were lysed and Gαi which has not been ADP-ribosylated during the intoxication with PT was ADP-ribosylated and biotin-labeled *via* the incubation with PTS1 and biotin-labeled NAD^+^. Subsequently, the biotin labeled Gαi was detected *via* Western Blot, while Hsp90 served as control for equal protein loading. The bar graph (*A*) shows the quantification of Western blot signals from three independent experiments, while (*B*) shows blots of a representative experiment. The intensity values of the bar graph are given as x-fold of the untreated control (Con), normalized to Hsp90, mean ± SEM (n = 3 values from three independent experiments). *C*, The 488-labeled PT (500 ng/ml) was preincubated for 15 min at 4 °C on CHO-K1 cells to enable binding of PT to cells but no internalization. Subsequently, cells were washed by centrifugation, and different concentrations of α_1_AT or the respective amount of solvent (H_2_O) were added to the cells and incubated for further 15 min at 4 °C. Cells were *left* untreated as control (Con). After that, cells were washed by centrifugation and 488-labeled PT bound to cell surfaces was measured using flow cytometry. Values of median are given as x-fold of the untreated control (Con), mean ± SEM (n = 9 values from three independent experiments). *A* and *C*, significance was tested using one-way ANOVA followed by Šídák’s multiple comparison test (*A*) or Dunnett’s multiple comparison test (*C*) and refers to samples treated with PT only (*A*, *C*) (∗*p* < 0.1, ∗∗*p* < 0.01, ∗∗∗*p* < 0.001, ∗∗∗∗*p* < 0.0001, ns not significant). α_1_AT, α_1_-antitrypsin; FCS, fetal calf serum; Gαi, α-subunit of inhibitory G protein; PT, pertussis toxin.

To explore whether the sequence of PT and α_1_AT application to cells influences the inhibition, we analyzed cell intoxication by PT after introducing PT and α_1_AT in varying orders (Fig. 8). Preincubation of cells with α_1_AT prior to PT treatment demonstrated that α_1_AT inhibition of PT intoxication occurred only when cells were not washed in between incubations. Further, α_1_AT did not significantly inhibit PT intoxication if cells were preincubated with PT prior to α_1_AT application independent of whether cells were washed in between, suggesting that bound PT cannot be inhibited sufficiently by α_1_AT. For control, cells were treated with α_1_AT and PT that were preincubated together before addition to cells, or α_1_AT and PT were added directly to the cells as it was done in the previous experiments. Under these conditions a significant inhibition was observed. Hence, α_1_AT displays a limited capacity to chase PT, if washed away or if PT is added first to cells, α_1_AT is not able to fully prevent intoxication of cells compared to conditions where α_1_AT is constantly present or preincubated with PT before addition to cells. In addition, the binding of PT, analyzed by flow cytometry, confirmed that α_1_AT displacement of cell-bound PT is limited when cells are preincubated with PT prior to α_1_AT application (Fig. 8*C*). These results suggest that interaction and, therefore, inhibition or inactivation of PT by α_1_AT occurs predominantly in solution prior to binding of PT to cells. This also indicates that PT is not covalently or irreversibly bound to cell surfaces.

### *In silico* analysis of the interaction between PT and α_1_AT

**Figure 9:**
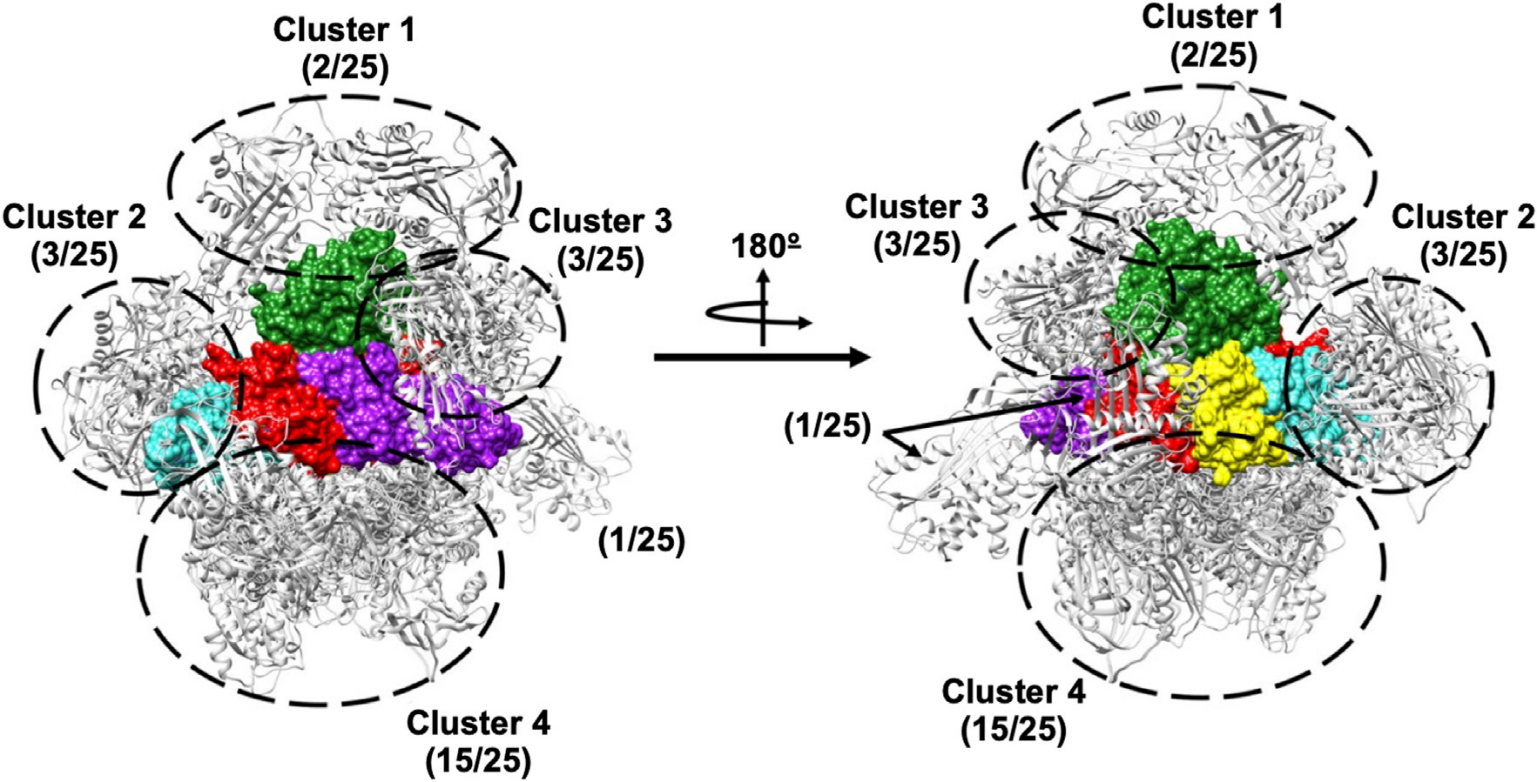
**Docking analysis.** Clusters obtained from the docking procedure with the servers GRAMM-X, HawDock, and AlphaFold3. α_1_AT is represented in *gray carton* and pertussis toxin in colored surfaces, S1, *green*; S2, *turquoise*; S3, *purple*; S4, *red*; S5, *yellow*. α_1_AT, α_1_-antitrypsin.

To study the potential interaction mechanism between α_1_AT and PT, we conducted several theoretical analyses. Initially, blind docking was performed, allowing α_1_AT to explore the entire surface of PT in search of the optimal interaction conformations. To ensure the maximum exploration of the PT’s surface, three different web servers, each with its own search algorithm, were used (see Experimental procedures, “In silico analysis of the PT-α1AT interaction”). This resulted in the identification of four representative clusters. Clusters one and three showed some interactions with subunit S1; however, these have a low representation, with only two and three results from the 25 obtained indicating a low probability of occurrence. On the other hand, cluster two interacts only with subunit S2 but also with low results (3/25). Finally, Cluster for was the most predominant (15 out of 25 results), indicating that there is a higher probability that α_1_AT interacts with PT by this region (Fig. 9). Notably, conformations obtained in this cluster exclusively interacted with the pentameric ring of PT and did not engage with the catalytic subunit.

**Figure 10:**
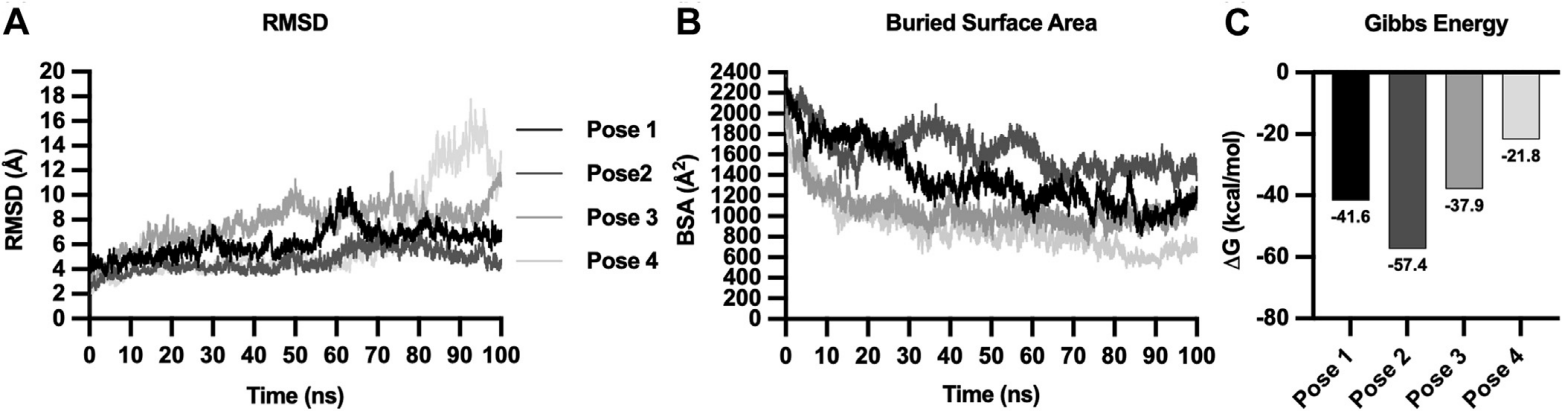
**Molecular dynamics simulations for most stable α**_**1**_**AT-PT complexes obtained from docking.***A*, root mean square deviation analysis in the function of time. *B*, buried surface area of α_1_AT with PT in the function of time. *C*, Gibbs energy calculations. α_1_AT, α_1_-antitrypsin; PT, pertussis toxin.

To identify the most probable binding conformation of α_1_AT with PT, 11 poses were extracted from cluster four (Table 1). Since the scores for conformations obtained from different servers are not comparable, we rescored them using the Prodigy server (35). This allowed us to establish a hierarchical order from the most stable complexes to the lower, based on the binding energy obtained with this server. The conformations obtained with the GRAMM-X server were consistently ranked as the most favorable, with high differences in results from HawDock and AlphaFold3 (Table 1). According to these results, the stability of the most stable complexes obtained from docking was evaluated by molecular dynamics simulations. The most probable binding conformation was proposed based on the analysis of RMSD, buried surface area (BSA), and Gibbs energy. The second-best conformation (pose two), according to the Prodigy scoring function, exhibited the highest stability among the evaluated conformations (Fig. 10). Pose two demonstrated the lower RMSD value (∼4 Å) with minimal fluctuations and a fast saturation of the curve (Fig. 10*A*). Additionally, it featured the higher BSA of α_1_AT with the PT (∼1600 Å^2^), remaining unchanged after 60 ns simulation (Fig. 10*B*). Finally, Pose two showed a lower Gibbs energy, indicating a higher stability of this complex (Fig. 10*C*).

**Table 1:** Rescoring using the Prodigy server of the 11 representative conformations obtained from the fourth cluster

Pose	Server	Name	Prodigy (kcal/mol)
1	GRAMM-X	result4	−19
2	GRAMM-X	result3	−17.9
3	GRAMM-X	result1	−17.5
4	GRAMM-X	result5	−14.3
5	AlphaFold3	model1	−11.7
6	HawDock	model9	−10.3
7	HawDock	model2	−9.5
8	AlphaFold3	model0	−9.3
9	HawDock	model5	−8.9
10	AlphaFold3	model3	−8.6
11	AlphaFold3	model2	−7.9

**Figure 11:**
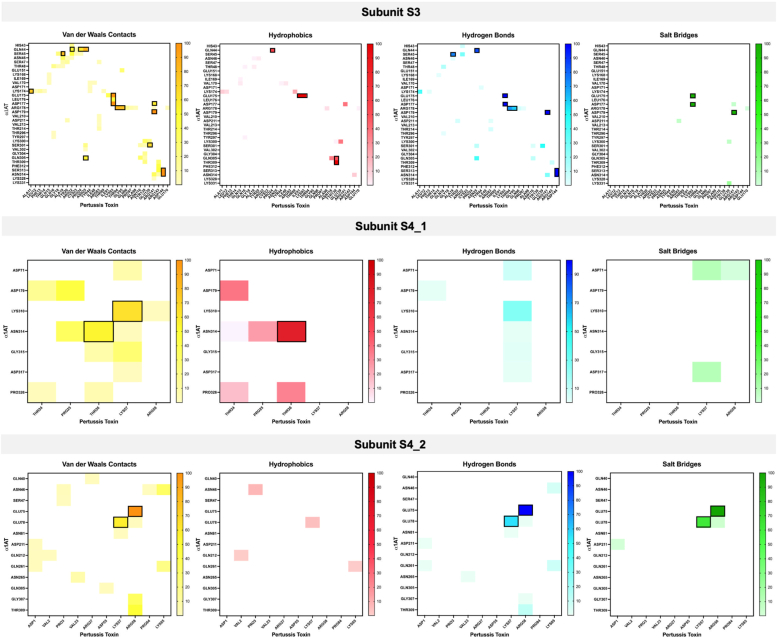
**Energy decomposition of the interaction per residue in the last 10 ns of simulations.** van der Waals contacts, hydrophobic interactions, hydrogen bonds, and salt bridges are in *yellow*, *red*, *blue*, and *green* colors, respectively. *Colored squares* represent contact points between α_1_AT and PT. The stronger the color, the longer the residues interacted. Enclosed in squares are those residues interacting at least 50% (5 ns) of the time of the simulations. α_1_AT, α_1_-antitrypsin; PT, pertussis toxin.

To find the most important residues and the forces driving the stability of the complex, the interactions between α_1_AT and PT during the last 10 ns of the simulation were studied. As illustrated in Fig. 11, α_1_AT exhibited the largest number of interactions with subunit three (S3), contacting at least 31 amino acid residues from pertussis toxin. With both subunits S4 it also displayed some interactions contacting five and ten residues from S4_1 and S4_2, respectively. One interaction was observed with residue HIS49 from subunit five (S5); however, it was for a very short period (0.82 ns). No interactions were detected with subunit one (S1). Eighteen residues from α_1_AT present occupancies of interaction with PT higher than 50% of the simulation time, indicating a high importance for complex stabilization. The energy decomposition showed four main driving forces for the interaction of α_1_AT with PT, van der Waals contacts, hydrophobic interactions, hydrogen bonds, and salt bridges. For subunits S3 and S4_2, electrostatic interactions (hydrogen bonds and salt bridges) seem to play an important role in the stabilization of the complex, with residues interacting almost all the time during the simulations. Among these residues can be highlighted GLN44, SER45, GLU175, ASP177, ARG178, ASP179, SER313, and ASN314 for interaction with S3 and residues GLU75 and GLU78 for the interaction with S4_2. Conversely, for subunit S4_1, nonpolar interactions (van der Waals, hydrophobic) are more essential than electrostatics ones. Here, only two residues showed interactions with more than 50% of the evaluated time LYS310 and ASN314, and these were through weak forces (van der Waals, hydrophobic).

To further explore the importance of residues presenting more than 50% occupancy in the stability of the complex, an *in silico* alanine scanning was performed (Fig. S8). For this, the server Mutabind2 was used following three different approaches (1), mutate all residues for alanine at the same time (2), separate the residues in three specific clusters and mutate all the residues from each cluster; cluster 1: E75, E78, T309, K310, and S313; cluster 2: K174, E175, L176, D177, R178, and D179; and, cluster 3: Q44, S45, S301, and Q305. The residue N314 showed a spatial disposition in the borderline between cluster 1 and cluster 2; due to this, both possibilities were evaluated (Fig. S8) (https://www.gov.uk/government/publications/health-protection-report-volume-18-2024/hpr-volume-18-issue-1-news-1-february-2024). Each residue was mutated for alanine, and its contribution to the complex stability was evaluated individually.

Table S1 shows the ΔΔG values obtained for each approach. ΔΔGbind (kcal mol-1) is the predicted change in binding affinity induced by a mutation. Positive and negative signs correspond to destabilizing and stabilizing mutations, which are predicted to decrease and increase binding affinity correspondingly. The server classifies a mutation as deleterious if ΔΔG ≥ 1.5 or ≤ −1.5 kcal mol-1 (36).

As expected, the change of all residues at the same time for alanine leads to a significant destabilization of the complex, reinforcing their importance in the interactions of α_1_AT with PT. The analysis by clusters highlighted the importance of N314 in the stabilization of the complex. When this residue is included in cluster 1 the change of residues for alanine leads to a significant increase in the ΔΔG. However, when it is not included, the predicted binding affinity shift induced by mutations does not reach the threshold value. On the other hand, mutations in cluster two always led to the destabilization of the complex. Notably, when residue N314 is included, the destabilization is higher. Similar to cluster two, mutations in cluster three led to a drop in stability.

In the evaluation of the individual contribution to the stability, N314 comes to light again, being the only residue that showed a ΔΔG > 1.5 kcal/mol. However, other residues showed values close to the threshold as K174 (1.36 kcal/mol), E175 (1.48 kcal/mol), D177 (1.46 kcal/mol), and D179 (1.39 kcal/mol). Notably, all these residues belong to cluster two and are mostly involved in the electrostatics interaction with subunit S3. Interestingly, residues belonging to cluster three do not show significant ΔΔG individually; however, as mentioned above, the mutation of the whole cluster did lead to the destabilization. These residues are mainly involved in interaction with residues from S3 either by polar (Q44 and S45) or nonpolar (S301 and Q305) interactions. Finally, the residues of group one do not present a significant contribution to the stability either individually or by the mutation of the entire cluster. It is worth highlighting that some residues from this cluster are mostly involved in the interaction with subunits S4_1 (K310) and S4_2 (E75 and E78).

Collectively, all results presented above suggest that the potential mechanism of α_1_AT inhibition against PT may involve interfering with the interaction of the toxin with cellular receptors rather than affecting its catalytic activity. Additionally, this inhibition seems to occur mainly by the interaction of α_1_AT with subunit S3 through electrostatic interactions facilitated by residues N314, K174, E175, D177, and D179.

### α_1_AT interacts with PT but not with PTS1

**Figure 12:**
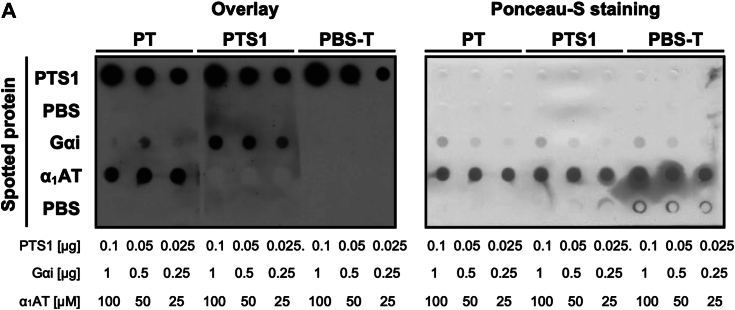
**Interaction of α**_**1**_**AT with PT and PTS1 *in vitro.****A*, decreasing concentrations of PTS1 (antibody control), Gαi, and α_1_AT were vacuum aspirated onto a nitrocellulose membrane using the Dot blot system. PBS was aspirated as a control. Subsequently, the membrane was stained with Ponceau-S (*right*), blocked, and cut for incubation with the overlay samples, PT (200 ng/ml = 0.001904 μM), PTS1 (200 ng/ml = 0.0007616 μM), and PBS-T as control. The amount of spotted PTS1 is below the detection limit of Ponceau-S staining. Bound PTS1 was detected using an antibody against PTS1 (*left*). A representative blot is shown of at least three independent experiments (n = 3). α_1_AT, α_1_-antitrypsin; Gαi, α-subunit of inhibitory G protein; PT, pertussis toxin.

Prompted by the findings revealed by molecular docking and dynamics analysis, we investigated whether α_1_AT directly interacts with PT or PTS1. Therefore, α_1_AT was vacuum-aspirated onto a membrane and overlaid with PT or PTS1 in solution. Bound PT/PTS1 was detected by a specific antibody. Using this approach, α_1_AT was determined to directly interact with the PT holotoxin but not with the PTS1 enzyme subunit (Fig. 12*A*). Gαi was spotted as a known interaction partner of PTS1. The His-tag of recombinant Gαi had no effect on binding to PTS1 (Fig. S9). As expected, PTS1 showed interaction with its specific substrate Gαi, indicating that the interaction of α_1_AT with PT is specific and is most likely facilitated by the B-subunits of PT as predicted by the molecular modeling data. PTS1 was spotted to confirm the activity of the specific antibody. PBS was spotted to rule out nonspecific membrane binding of the overlay protein. Overlay with PBS-T was performed to exclude nonspecific binding of the antibodies. It was shown before that the *Clostridioides (C.) difficile* AB-toxin TcdB forms aggregates in the presence of the antimicrobial peptide α-defensin-6 and thereby inhibits the binding of TcdB to cells and thus intoxication (37). Therefore, the effect of α_1_AT on aggregate formation with PT or TcdB was tested by incubating the toxins with α_1_AT in reaction tubes followed by centrifugation. The supernatant and the pellet fraction were subjected to immunoblot analysis. Results in Fig. S9*B* show that PT was detected in the supernatant and also in the pellet fraction without the addition of α_1_AT. Detection in the pellet fraction might be due to unspecific binding of PT to the tube. This distribution was not altered in the presence of α_1_AT. TcdB was detected as expected in the supernatant and after incubation with α-defensin-6 in the pellet due to aggregation. In the presence of α_1_AT, TcdB was still predominantly detected in the supernatant, and only a weak signal was observed in the pellet fraction. These results suggest that PT and TcdB show no or only minor formation of aggregation in the presence of α_1_AT.

### α_1_AT ameliorates *B. pertussis*-induced leukocytosis

**Figure 13:**
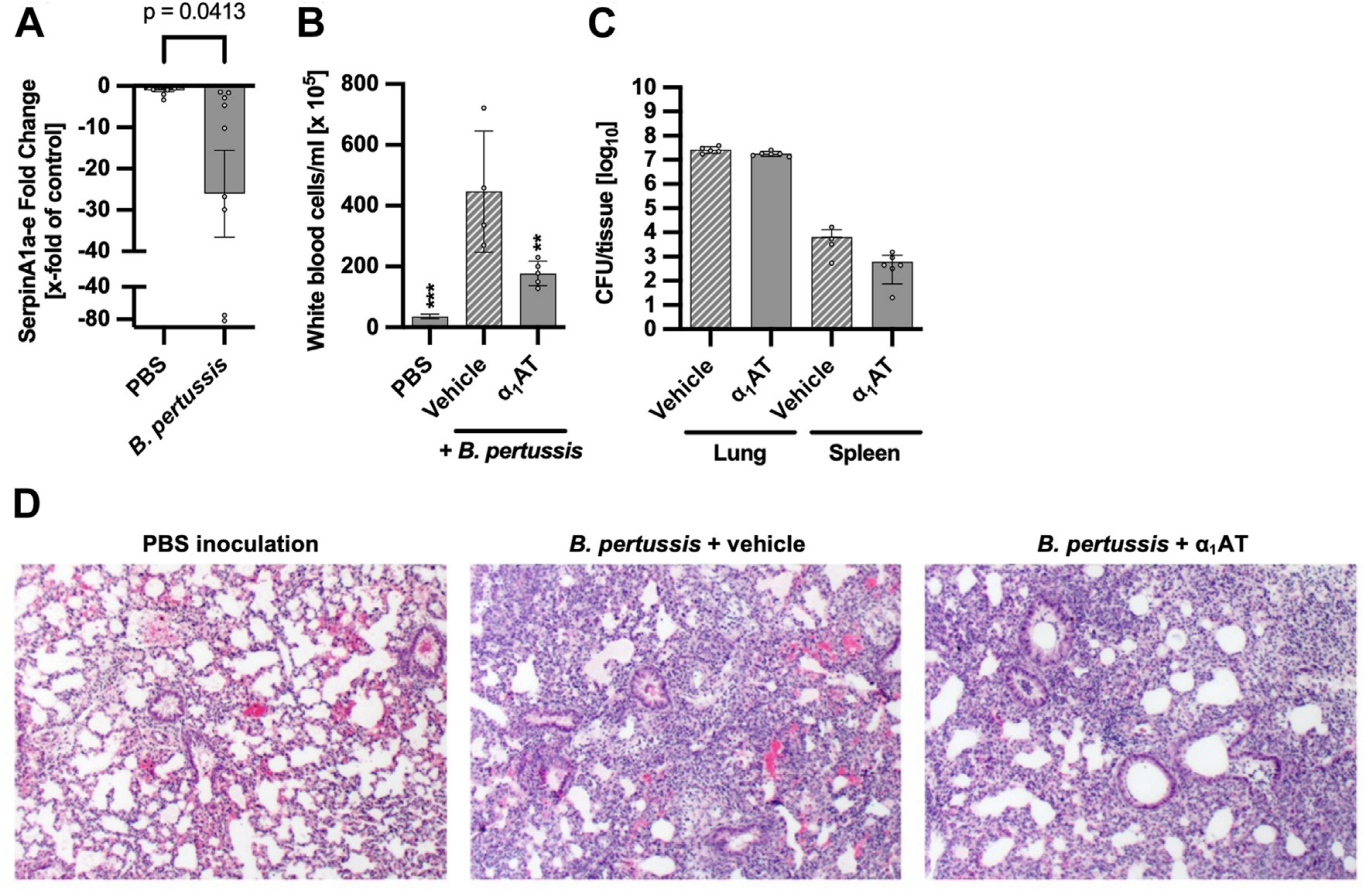
**α**_**1**_**AT levels correlate with *B. pertussis* infection and leukocytosis.***A*, SerpinA1a-e, the murine α_1_AT genes, were quantified in RNA isolated from the lungs of infant mice at 8 days post inoculation by qRT-PCR. Data represent fold changes in serpinA1a-e expression relative to control PBS-inoculated mice and normalized to a housekeeping gene. Bars graphs depict a representative group of n ≥ 4 mice for experiments performed two times. Values are given as mean ± SD. *B* and *C*, mice were aerosolized with a bacterial inoculum at absorbance 1.0 (6 × 10ˆ9 CFU/ml confirmed) and treated with 50 mg/kg α_1_AT from human plasma at a concentration of 10 mg/ml *via* intraperitoneal injection every second day from day 0 (immediately after infection). Harvest was done at 8 days post infection. *B*, white blood cell count was determined in peripheral blood. Values are given as mean ± SEM. *c*, colony forming units (CFU) in the lung and spleen were determined. Values are given as mean ± SEM. *d*, lung histopathology was evaluated from hematoxylin and eosin stained sections. *A*, *B*, and *C*, significance was tested using (*A*, *C*) unpaired *t* test or (*B*) one-way ANOVA followed by Dunnett’s multiple comparison test and refers to *B. pertussis* infected mice treated with vehicle (∗*p* < 0.1, ∗∗*p* < 0.01, ∗∗∗*p* < 0.001, ∗∗∗∗*p* < 0.0001, ns not significant). The *p*-value for unpaired *t* test is reported as indicated, when lower than 0.05. α_1_AT, α_1_-antitrypsin; qRT-PCR, quantitative reverse transcription PCR.

Infants are uniquely susceptible to severe outcomes of *B. pertussis* infection such as leukocytosis and pulmonary hypertension (38). These severe pathologies are PT-mediated, and risk factors associated with death (39, 40, 41, 42). In addition, α_1_AT deficiency correlates with inflammatory pathology in the lungs and increased susceptibility to respiratory infection (43). Infant C57BL/6 mice recapitulate pertussis-associated leukocytosis and pulmonary inflammation when infected with *B. pertussis* (41). As such, this mouse strain was used to determine the effect of *B. pertussis* infection on α_1_AT expression and the impact of exogenous α_1_AT administration in infant disease. While humans have only one gene that codes α_1_AT, SerpinA1, mice have up to six paralogs. The mouse strain used in this study, C57BL/6 mice, express five of these paralogs, SerpinA1a-e. Of these, the proteins translated from SerpinA1a and Serpina1b are the only ones which inhibit neutrophil elastase, the major substrate target for human α_1_AT (44). Seven-day-old C57BL/6 mice were inoculated with *B. pertussis* or vehicle (PBS), and, at a time point when infants mice begin to succumb to infection (8 days post inoculation (41)), the expression levels of the murine genes that code for α_1_AT, serpinA1a-e, were determined by quantitative reverse transcription PCR on whole lung homogenates. At this time point, infected mice had 1.76 × 10^8^ – 1.66 × 10^9^ colony forming units of *B. pertussis* per left lung lobe and demonstrated a significant downregulation of serpinA1a-e compared with age-matched PBS-inoculated control mice (Fig. 13*A*). These data indicate that infection with *B. pertussis* can limit the expression of pulmonary α_1_AT and may thereby prevent α_1_AT-mediated inhibition of PT and promote α_1_AT deficiency-associated airway inflammation. Future studies will investigate the mechanism and impact of this downregulation and examine the impact of *B. pertussis* on liver-derived α_1_AT. Next, the effect of exogenous α_1_AT treatment in *B. pertussis* infected infant mice was investigated to determine α_1_AT-mediated neutralization of PT activity *in vivo*. Mice were inoculated with *B. pertussis* and treated with α_1_AT by intraperitoneal injection every second day from day 0. After eight days, a white blood cell count was performed to assess leukocytosis, a hallmark of PT activity *in vivo* (41, 45), and lung histopathology, and colony-forming units in the lung and spleen were evaluated. Infant mice infected with *B. pertussis* and treated with vehicle PBS displayed significantly higher numbers of circulating white blood cells in comparison to control uninfected PBS-inoculated mice. While mice infected with *B. pertussis* and treated with α_1_AT displayed a significant reduction in leukocytosis in comparison to vehicle-treated *B. pertussis*-infected mice (Fig. 13*B*). This reduction in leukocytosis was independent of effects on bacterial load, as the numbers of bacteria in the primary site of infection, the lungs, or the site of bacterial dissemination, the spleen was unchanged (Fig. 13*C*). In addition, α_1_AT treatment caused no change to *B. pertussis*-induced lung pathology in comparison to *B. pertussis*-infected and vehicle-treated mice (Fig. 13*D*). Both groups of infected mice displayed a diffuse recruitment of immune cells and consolidation of alveolar tissue which was not observed in uninfected PBS-inoculated mice (Fig. 13*D*), indicating that α_1_AT does not protect against *B. pertussis*-induced airway pathology. This finding is consistent with previous studies demonstrating that, unlike adult mice, *B. pertussis-*induced airway pathology in infant mice is not caused by PT (41). Taken together, these data provide the first evidence that α_1_AT administration may be a valid approach for the treatment of PT-mediated pertussis manifestations in infants and support the need for further studies to comprehensively evaluate and optimize α_1_AT treatment strategies for this severe disease.

## Discussion

Pertussis remains a significant global health concern despite vaccination efforts (1, 2). The disease poses substantial morbidity and mortality, particularly among infants and young children. The increasing incidence of pertussis cases worldwide underscores the urgency for effective therapeutic interventions (1, 2). However, the lack of specific treatment options against pertussis presents a critical challenge in managing and mitigating its impact. Presently, the management of pertussis predominantly relies on supportive care and early antibiotic treatment to reduce transmission. However, no targeted therapeutics exist to combat the primary pathophysiological mechanisms of the disease. This deficiency highlights an urgent need to explore alternative therapeutic avenues to address the limitations of current treatment strategies.

This study identified α_1_AT as a novel inhibitor of PT by screening of a hemofiltrate peptide/protein library. α_1_AT reduces intoxication of cells with PT by directly interacting with PT, thereby impeding its binding to cells. Most likely, this direct interaction is driven by interaction of α_1_AT with the pentameric B-subunit of PT. Consequently, this interaction prevented PT’s cellular uptake and subsequent cellular reactions (Fig. S10). Using a neonatal *B. pertussis* infection model, we also demonstrated that α_1_AT significantly mitigates leukocytosis, a hallmark of severe pertussis associated with PT-expression. Additionally, our study revealed a significant reduction in α_1_AT expression in *B. pertussis* infected mice, shedding light on the potential implications of decreased α_1_AT levels during pertussis infection. These findings provide a proof of concept and underscore α_1_AT’s promising therapeutic potential in countering PT-mediated pertussis pathogenesis. However, further *in vivo* experiments are required to elaborate on required doses, application form, and influence of α_1_AT’s effect on PT-mediated pertussis pathogenesis in organs such as the lungs, liver, and spleen.

α_1_AT plays a crucial role in regulating protease activity and modulating inflammatory responses. It acts as a protective agent for lung tissue, shielding it from the harmful effects of enzymes released by immune cells, notably neutrophil elastase (46). Pertussis-induced lung pathology involves excessive inflammation and tissue damage, making α_1_AT an attractive therapeutic agent due to its antiinflammatory and tissue-protective properties in addition to its anti-PT effects. In healthy individuals, plasma concentrations of α_1_AT range between 0.9 and 2 mg/ml (corresponding to ∼ 17–38 μM) (47). Interestingly, during episodes of acute inflammation, this concentration can surge by 4 to 5 fold, indicating α_1_AT’s crucial role as acute phase protein in combating or preventing inflammation-induced tissue damage (47). The concentrations of α_1_AT span from 10 to 40 μM in alveolar interstitial fluid and approximately 2 to 5 μM in alveolar extracellular lining fluid (48, 49, 50). An inhibiting effect of α_1_AT against PT-intoxication of cells was observed at concentrations starting from around 30 μM α_1_AT, falling within the concentration ranges found in healthy individuals. However, the extent of this inhibiting effect was increased with higher concentrations. This study examined concentrations up to 100 μM, revealing the most robust impact. In our *in vivo* studies mice were administered with human α_1_AT at a concentration of 10 mg/ml, with each mouse receiving volumes corresponding to a dose of 50 mg/kg of mouse weight. In other studies involving humans, doses of 120 mg/kg and even 250 mg/kg were administered without observing side effects (51, 52). Hence, while α_1_AT administration reduced *B. pertussis-*induced leukocytosis but failed to protect against *B. pertussis-*induced airway pathology in the study presented here, we cannot rule out the potential for higher doses of α_1_AT to ameliorate this phenotype. Evaluation of the therapeutic potential of higher doses of α_1_AT will be the subject of future studies.

Of note, results from the murine infection model indicate that *B. pertussis* infection results in decreased pulmonary α_1_AT expression, potentially facilitating PT to exert its toxic effects more effectively in infected individuals. To our knowledge, no data are available regarding the α_1_AT expression in humans during *B. pertussis* infection. Investigating α_1_AT expression in human subjects amid *B. pertussis* infection could provide compelling insights into its role, possibly paralleling findings observed in murine models. If *B. pertussis* infection causes downregulation of α_1_AT expression in the human host to increase severity of PT effects, this would give novel insights on pertussis pathogenesis. In addition, due to its natural function as acute phase protein in humans, signaling mechanisms of systemic or local upregulation of α_1_AT expression are well described in the context of inflammation, while downregulation of α_1_AT expression is poorly understood (53). Hence, our results provide the base for further research into details of α_1_AT expression regulation upon *B. pertussis* infection.

α_1_AT was also identified as an inhibitor of SARS-CoV-2 through screening a peptide/protein library derived from human bronchoalveolar lavage (54). However, the inhibitory mechanism differs between SARS-CoV-2 and PT. In the case of SARS-CoV-2, α_1_AT inhibits TMPRSS2, a cell surface protease responsible for priming the spike protein of the virus. Consequently, this impedes the SARS-CoV-2 spike-driven cell entry, offering protection against infection (54), highlighting the broader role of α_1_AT in combating viral infections, as well as bacterial toxins. In the case of SARS-CoV-2, inhibition of TMPRSS2 is mediated by two amino acid residues of α_1_AT, LEU353, and ALA355. In our study, the identified amino acid residues of α_1_AT that were involved in interaction with the B-subunits of PT differed and included GLN44, SER45, GLU175, ASP177, ARG178, ASP179, SER313, ASN314, GLU75, GLU78, LYS310, and ASN314. As such, α_1_AT was identified as inhibitor of pathogens causing respiratory diseases where the mechanism of inhibition involves direct interaction of either TMPRSS2 or PT with α_1_AT, different amino acid residues of α_1_AT seem to be responsible for driving the interaction. In addition, the amino acid residues mentioned above are not part of α_1_AT’s reactive center loop bearing key residues (MET358-SER359), which are responsible for the cleavage of target proteases (47, 55). This further supports the hypothesis that the protease activity of α_1_AT is not relevant for the inhibition of PT, since in our experiments another protease inhibitor of the serpin family, antithrombin, did not inhibit PT. To validate that the amino acid residues of α_1_AT identified in this study are indeed important for interaction, mutating them would be a valid approach for further studies. In addition, we recently demonstrated that α_1_AT acts as an inhibitor of several other bacterial toxins, including *Clostridium botulinum* C2 toxin, *Corynebacterium diphtheriae* diphtheria toxin (DT), and *Bacillus anthracis* fusion toxin (56). Specifically, we showed that α_1_AT inhibits C2 toxin by inhibiting its binding to cells and by inhibiting the enzyme activity of its enzymatic active domain, C2I *in vitro*. Since these toxins require proteolytic activation of their B-subunits for intoxication, it was hypothesized that α_1_AT may inhibit these toxins by targeting the proteases required for their activation. However, our findings revealed that α_1_AT also inhibits proteolytically activated forms of both diphtheria toxin (nicked DT) and the B-component of anthrax toxin, PA63. These results suggest that the anti-protease activity of α_1_AT, as well as its potential inhibition of furin, may play a negligible role in our observations. Instead, we speculate that α_1_AT’s inhibition of these bacterial toxins may be driven by structural similarities or common amino acid compositions shared among the toxins tested.

Commercially available preparations of human α_1_AT, such as Prolastin used in this study as well as others, are obtained through purification of donated blood and have been approved for the treatment of patients with genetic α_1_AT deficiency for decades. α_1_AT preparations are used for intravenous substitution therapy to manage persistent and unregulated tissue degradation primarily in the lower respiratory tract caused by α_1_AT deficiency (32). α_1_AT can also be administered through inhalation, allowing for significantly higher doses than those typically used for routine deficiency treatment (57). Doses as high as 250 mg/kg exhibited a fivefold increase in serpin concentration within the epithelial lining fluid of the lung without inducing adverse effects (51). While primarily indicated for α_1_AT-deficiency, the multifaceted nature of α_1_AT’s antiinflammatory properties prompts exploration into its potential repurposing for pertussis management. The established safety profile and administration protocols of Prolastin render it an attractive candidate for repurposing studies in pertussis treatment. Certainly, future studies should focus on conducting animal studies, such as those in the established baboon model (58), to assess the potential impact of α_1_AT on the course of pertussis infection. Additionally, well-designed clinical trials are needed to evaluate the efficacy, optimal dosing, and long-term outcomes of α_1_AT therapy in pertussis patients. Investigations into potential biomarkers for response to treatment and the identification of any adverse effects specific to this new application are also essential to ensure safe and effective repurposing of the drug.

## Experimental procedures

### Hemofiltrate peptide/protein library and chromatographic purification of the active molecule

#### Generation of peptide/protein library from hemofiltrate

Hemofiltrate (150–250 l/week) was obtained from patients with organ failure and sepsis at the Anesthesiology and Intensive Medicine Clinic University Medical Center Ulm, Germany (Human HF was collected from multiple donors exhibiting the patient’s written informed consent prior to inclusion in the study and based on the positive vote 91/17 approved by the Ethics Commission of University of Ulm. The study abides by the Declaration of Helsinki principles.). Patients were subjected to arteriovenous hemofiltration using ultrafilters with a cutoff of around 30 kDa. Blood filtration was driven by a transmembranous pressure gradient of 60 to 100 mm Hg (1 mm Hg = 133.3 Pa) at a flow rate of 250 to 350 ml/min. Twenty to thirty L of filtrate were obtained per patient and treatment.

The sterile filtrate (1000 l) was diluted with deionized water to a conductivity value below 8 mS/cm, conditioned to pH 2.7 using hydrochloric acid (HCl), and then loaded at a flow rate of 65 ml/min onto a 40 × 15 cm strong cation-exchanger Fractogel TSK SP 650(M) (Merck), followed by a washing step using 0.01 M HCl. At the outlet of the cation-exchange column, a reversed-phase column 15 × 6 cm (Amberlite XAD, Supelco) was coupled to capture the peptides not retained in the CEX column. Stepwise elution of bound peptides from the CEX column was performed with the following buffers in increasing order of pH: E1, 0.1 M citric acid, pH 3.6, σ = 2.9 mS/cm; E2: 0.1 M acetic acid + 0.1 M sodium acetate, pH 4.5, σ = 4.0 mS/cm; E3: 0.1 M disodium hydrogen phosphate, pH 7.4, σ = 6.7 mS/cm and E4: 0.5 M ammonium carbonate, pH 9.0, σ = 6.7 mS/cm. The Amberlite XAD column was washed with 25%, 50%, 75%, and 100% acetonitrile (J. T. Baker) to generate pools E5, E6, E7, and E8, respectively.

Following batch elution, every pool (10 l) was loaded onto a reversed-phase column Sepax Poly RP-300 of dimensions 25 × 5 cm and particle size 10 μm at a flow rate of 150 ml/min. After sample application, the column was washed with two column volumes of 95% A (water with 0.1% TFA, Merck) and 5% B (acetonitrile, 0.1% TFA), and the separation was performed by gradient elution from 5% to 100% B in 55 min, at 100 ml/min. Fractions of 100 ml were collected. After the separation, the column was reequilibrated with two column volumes of 95% A and 5% B. Aliquots representing one percent of every chromatographic fraction were sampled in deep well microtiter tubes in a 96-well format using a pipetting robot, then lyophilized and stored at −20 °C. The remaining of each fraction was stored at −80 °C.

#### Bioassay-guided chromatographic purification of the biologically active molecule

The dry material corresponding to the active fraction was dissolved in 10 ml of 10% acetic acid and fractionated by reversed-phase HPLC on a Luna C18 column (Phenomenex) of dimensions 21.2 × 250 mm and particle size of 5 μm. The separation was performed at a flow rate of 12.23 ml/min using the gradient program (min/% B) 0/5, 5.09/5, 28.71/25, 49.55/50, and 70.39/100, being A, 0.1% TFA in water, and B, 0.1% TFA in acetonitrile. Elution was monitored online at 280 nm. Fractions were collected every minute and dried in a SpeedVac vacuum concentrator.

The active fractions from the previous purification step were dissolved in 1 to 2 ml 10% acetic acid and fractionated by reversed-phase HPLC on Aeris PEPTIDE XB-C18 column (Phenomenex) of dimensions 10 × 250 mm and particle size of 5 μm. The separations were performed at a flow rate of 3 ml/min using the gradient program (min/% B) 0/5, 5/25, 65/55, and 70/80, being A, 0.1% TFA in water, and B, 0.1% TFA in acetonitrile. Elution was monitored online at 214 nm.

### Identification of the biologically active molecule by mass spectrometry

The samples were reduced with 5 mM DTT for 20 min at room temperature, carbamidomethylated with 50 mM iodoacetamide for 20 min at 37 °C, and digested with trypsin (Thermo Fisher Scientific, 900, 589), at a 1:50 ratio (enzyme:protein) for 16 h at 37 °C. A 15 μl-aliquot of every sample was subjected to liquid chromatography with tandem mass spectrometry analysis on an Orbitrap Elite system an Orbitrap Elite Hybrid mass spectrometry system (Thermo Fisher Scientific) online coupled to an U3000 RSLCnano system (Thermo Fisher Scientific) as previously described (59).

Database searches were performed using PEAKs XPro (PEAKs studio 10.6), http://www.bioinfor.com/peaks-studio (60). For peptide identification, MS/MS spectra were correlated with the UniProt human reference proteome set, www.uniprot.org. Carbamidomethylated cysteine was considered as a fixed modification along with oxidation (M) and deamidation (NQ) as variable modifications. False discovery rates were set on the peptide level to 1%.

### Compounds and reagents

The *B. pertussis* toxin (PT) used for all the experiments was purchased from Sigma-Aldrich, Merck. The native toxin TcdB from *Clostridium difficile* VPI 10463 was expressed and purified as described elsewhere (61). The recombinant proteins Gαi and PTS1 were expressed and purified as described earlier (62). The source of α_1_AT was the commercially available drug Prolastin purchased from Grifols. The tested antithrombin was the drug Kybernin P 500 purchased from CSL Behring. The peptide α-defensin-6 was purchased from PeptaNova.

### Cell lines

The materials for the cultivation of all cell lines were purchased from Gibco (Thermo Fisher Scientific) unless mentioned differently. The used cell lines included A549 cells (human lung adenocarcinoma cells; American Type Culture Collection) and CHO-K1 cells (Chinese hamster ovary cells strain K1; DSMZ). A549 cells were cultivated in Dulbecco's modified Eagle's medium, supplemented with 10% FCS, 1 mM sodium pyruvate, 0.1 mM nonessential amino acids and 100 U/ml penicillin and 100 g/ml streptomycin. CHO-K1 cells were cultivated in Dulbecco's modified Eagle's medium and HAM’s F12 (1:1), supplemented with 5% FCS, 1 mM sodium pyruvate, 0.05 mM nonessential amino acids and 100 U/ml penicillin and 100 g/ml streptomycin. Cells were cultivated under humidified conditions at 37 °C with 5% CO_2_ and trypsinized and reseeded every two to three days for at most 25 times. For intoxication experiments, cells were seeded in culture dishes one or two days before and treated in FCS-free media with toxins and the respective compounds.

### Sequential ADP-ribosylation of Gαi in toxin-treated cells

For the analysis of the ADP-ribosylation status of Gαi, cells were seeded in 24-well plates for one day before treatment with hemofiltrate library fractions, α_1_AT, water (solvent control), and PT in FCS-free medium. For screening experiments, samples from the hemofiltrate library, samples from chromatographic fractionations, and water (solvent control) were added directly with PT to cells. For selected experiments α_1_AT, water (solvent control), and PT were either preincubated for 15 min at room temperature before addition to cells or added directly to cells without preincubation. After 4 h of intoxication at 37 °C, cells were washed, and cell lysates were generated *via* freezing of plates. The cell lysates were collected in ADP-ribosylation buffer (0.1 mM Tris–HCl (pH 7.6), 20 mM DTT, 0.1 μM ATP, and protease inhibitor complete (Roche)). The sequential *in vitro* ADP-ribosylation of Gαi, which had not been modified during previous intoxication of cells with PT, was performed using recombinant PTS1 (95.7 nM). The reaction was labeled *via* biotin-labeled NAD^+^ (1 μM; R&D Systems) during the 40 min incubation at room temperature. After denaturation of the samples, gel electrophoresis, and Western blotting were performed. Sequentially ADP-ribosylated Gαi, which was previously biotin-labeled, was detected using streptavidin-peroxidase (Strep-POD, Sigma-Aldrich, Merck). Hsp90 (primary antibody from Santa Cruz Biotechnology) was detected as loading control and used to normalize Gαi signal intensity. For the quantification of signals, ImageJ software v.1.52.a (National Institutes of Health, Bethesda (NIH; https://imagej.net/ij/) was used.

### Cell viability assay

For the analysis of effects on cell viability CHO-K1 cells were seeded in 96-well plates and treated with α_1_AT, water (solvent control), and 20% dimethylsulfoxide as a positive control for cell death in FCS-free medium for 4 or 24 h. The cell viability was assessed using the CellTiter 96 Aqueous One Solution cell proliferation assay (MTS assay, Promega), which was added to the cells and incubated for 45 min at 37 °C. The absorbance was measured using a plate reader at 490 nm.

### Enzyme activity assay of PTS1 from cell lysates

For the analysis of the *in vitro* enzyme activity of PTS1, cell lysate from CHO cells was generated. Therefore, CHO cells were seeded in 10 cm culture dishes and grown for two to three days. Then, the cells were washed, and the culture dishes were frozen for lysis. Next, ADP-ribosylation buffer (0.1 mM Tris–HCl (pH 7.6), 20 mM DTT, 0.1 μM ATP, and protease inhibitor complete (Roche)) was added, and the cell lysate was collected in tubes. After centrifugation of the cell lysate at 10,000*g* for 1 min, the supernatant was transferred into a new tube, and the protein concentration was determined using the Nanodrop. Different concentrations of α_1_AT, water (solvent control), and 30 μg CHO cell lysate were mixed in ADP-ribosylation buffer. Subsequently, recombinant PTS1 (84 nM) and biotin-labeled NAD^+^ (1 μM; R&D Systems) were added for labeling of the ADP-ribosylation of Gαi during the 40 min incubation at room temperature. After that, the samples were subjected to gel electrophoresis and Western blotting. The ADP-ribosylated Gαi by PTS1 was detected using streptavidin-peroxidase (Strep-POD, Sigma-Aldrich, Merck). Hsp90 (primary antibody from Santa Cruz Biotechnology) served as loading control, and the signal quantification was performed using the ImageJ software v.1.52.a (NIH).

### Gel electrophoresis and Western Blotting

After sample preparation, Laemmli buffer (0.3 M Tris–HCl, 10% SDS, 37.5% glycerol, 0.4 mM bromophenol blue, 100 mM DTT) was added, and samples were heat-denatured at 95 °C for 10 min. For protein separation *via* gel electrophoresis, 8 or 12.5% acrylamide gels, depending on the size of the protein of interest, were used. The transfer of proteins from the gels onto nitrocellulose membranes was performed by semi-dry Western blotting and controlled by staining the membranes with Ponceau-S-staining (AppliChem GmbH). The membranes were blocked in 5% skim milk powder in PBS-T (PBS containing 0.1% Tween 20) for at least 30 min at room temperature, followed by washing steps in PBS-T. Next, incubation with primary and secondary antibodies in PBS-T separated by washing steps was performed. After the final washing steps, signals were detected using Pierce ECL Western blotting substrate (Thermo Fisher Scientific) and X-ray films (AGFA HealthCare).

### Binding analysis using flow cytometry

For the binding analysis based on flow cytometry, PT was labeled with DyLight 488 NHS Ester (Thermo Fisher Scientific) according to the manufacturer’s protocol using the Zeba Spin Desalting Columns (7K MWCO, Thermo Fisher Scientific) to remove excess dye. Next, cells were grown in a culture dish, detached using 25 mM EDTA in PBS, and resuspended in FCS-free medium. 488-labeled PT, α_1_AT, and water (solvent control) were preincubated for 15 min at room temperature or added directly to cells (1 × 10^5^ in 0.2 ml per sample) for 15 min at 4 °C to enable binding of PT to cells but no internalization. The samples were washed by centrifugation, and the cell fluorescence was measured using the BD FACS Celesta flow cytometer (Becton, Dickinson and Company) and the BD FACSDiva software 8.0.1.1 (https://www.bdbiosciences.com/en-de/products/software/instrument-software/bd-facsdiva-software). Using the blue laser (488 nm) 488-labeled PT was excited, while the emitted fluorescence was detected *via* the 530 nm (530/30) bandpass filter. Cell gating and data analysis was performed using Flowing Software v2.5.1. (Turku Centre of Biotechnology, Finland; https://bioscience.fi/services/cell-imaging/flowing-software/).

### Cellular binding and uptake analysis using fluorescence microscopy

For immunostaining and fluorescence microscopy experiments, cells were seeded and grown for 1 day in 18-well μ-slides (ibidi GmbH). Subsequently, cells were treated with α_1_AT or water (solvent control) and intoxicated in FCS-free medium with PT for the uptake analysis for 4 h at 37 °C or for the binding analysis for 40 min at 4 °C. After that, the cells were washed with PBS, fixed with 4% paraformaldehyde for 20 min, permeabilized using 0.4% (v/v) Triton X-100 in PBS for 5 min if required, and quenching was performed for 2 min in glycine (100 mM in PBS). Next, the cells were blocked for 1 h at 37 °C in PBS-T (PBS containing 0.1% Tween 20) containing 10% normal goat serum (Jackson ImmunoResearch) and 1% bovine serum albumin. After that, the incubation with primary antibodies for PTS1 (anti-PTS1, clone 63.1G9, sc-57639, Santa Cruz) and α_1_AT (Alpha-1-Antitrypsin antibody, Proteintech, Planegg-Martinsried) in blocking solution was performed for 1 h at 37 °C. The primary antibodies were detected *via* fluorescently labeled secondary antibodies in blocking solution for 1 h at 37 °C, and cell nuclei were stained for 5 min using Hoechst 33342 (1:10,000, Thermo Fisher Scientific). After staining, the slides were examined *via* microscopy using the BZ-X810 Keyence fluorescence microscope (Keyence Deutschland GmbH, Neu-Isenburg) and BZ-X800Viewer v1.3.0. The signals for PTS1 and Hoechst were quantified using the ImageJ histogram analysis.

### Uptake analysis using STED microscopy

For STED microscopy experiments the steps from seeding to incubation with the primary antibody were performed as described in 4.10. Secondary antibodies were incubated for 1 h at room temperature (anti-tb-aberrior STAR RED (Aberrior STED-1002–500UG), anti-ms-Atto594 (Sigma-Aldrich 76085-1ml-F), 1:1000, in 1:10 blocking buffer). Cells were then washed three times for 10 min with PBS to remove unbound secondary antibodies. Samples were stored at 4 °C until imaging. Just prior to imaging, cells were covered in 97% 2,2′-thiodiethanol.

STED images were taken using a custom-built dual-color three-dimensional STED microscope described in Osseforth *et al.* (63). For improved cross-talk reduction the emission filter in front of the 590 nm APD was changed to 623/24 BrightLine HC (AHF).

For sample illumination, a randomly polarized super-continuum laser source (repetition rate 1 MHz) was split into the excitation wavelengths of 568 nm and 633 nm and their respective depletion wavelengths of 710 nm and 750 nm. A typical excitation beam power of ∼0.8 μw and a depletion beam power of ∼1.3 mW were used during image acquisition. Confocal images were taken with a pixel size of 50 nm, at a dwell time of 200 μs per pixel, and a peak photon number of 160 to 200 counts. STED images were taken with a pixel size of 20 nm, at a dwell time of 300 μs per pixel, and a peak photon number of ∼150 counts. Image analysis was done using ImageJ. For better visualization Gaussian blur with sigma = 1.5 was applied.

### *In vitro* protein-protein interaction analysis by dot blot

For the *in vitro* analysis of protein-protein interactions, the dot blot system (Bio-Rad) was used. Therefore, decreasing concentrations of purified recombinant proteins, Gαi and PTS1 (antibody control), α_1_AT, and PBS (protein-free negative control) were vacuum aspirated onto a nitrocellulose membrane. Before blocking in 5% skim milk powder in PBS-T and cutting of the membrane, the transfer of proteins to the membrane was confirmed *via* the Ponceau-S-staining. After the washing steps in PBS-T, the overlay with PT or PTS1 in PBS-T, and PBS-T (negative control) for 1 h at room temperature was performed. This was followed by washing steps in PBS-T and incubation with the primary antibody for PTS1 (anti-PTS1, clone 63.1G9, sc-57639, Santa Cruz) and peroxidase-coupled anti-mouse antibody for the detection of bound PTS1.

### Precipitation assay

For the precipitation analysis, PT (100 ng) and TcdB (50 ng) and inhibitors, α_1_AT and α-defensin-6 (6 μM) were incubated for 30 min in PBS. After that, the samples were centrifuged for 20 min, 14,000 rpm at 4 °C. The supernatant was transferred into a new tube and the pellet was resuspended in PBS. The samples were subjected to gel electrophoresis and Western blotting. PT was detected using an anti-PTS1 antibody (anti-PTS1, clone 63.1G9, sc-57639, Santa Cruz) and TcdB was detected using an anti-TcdB antibody (Anti-*C. difficile* Toxin B antibody, Abcam).

### In silico analysis of the PT-α_1_AT interaction

The complexes PT-α_1_AT were obtained by blind docking using three different web servers, HawDock Server (http://cadd.zju.edu.cn/hawkdock/), GRAMM Docking Web Server (https://gramm.compbio.ku.edu/), and AlphaFold3 (https://alphafoldserver.com/) (64, 65, 66, 67, 68, 69). The coordinates for PT and α_1_AT were obtained from the Protein Data Bank (https://www.rcsb.org/) with the accession codes 1PTO and 1OPH, respectively. No restraints were applied to the docking, allowing the α_1_AT to explore the whole surface of PT. The best ten results for each server were analyzed and clustered. The region of PT with the highest number of clusters was assumed as the most probable region for the interaction of α_1_AT. From this cluster, the representative conformations were obtained using an RMSD cut-off of 2 Å, and its stability was evaluated by molecular dynamics simulations.

All simulations were performed using the NAMD 2.14 package (70) (https://www.ks.uiuc.edu/Research/namd/2.14/ug/node117.html) with the CHARMM36 force field (71, 72) (http://mackerell.umaryland.edu/charmm_ff.shtml). The TIP3P water model was used to generate explicit solvation conditions (73) and Newton’s equations of motion were integrated using the Verlet (leapfrog) algorithm (74). Periodic boundary conditions were applied in all directions, and the short-range van der Waals interaction cut-off was 1.2 nm. The particle mesh Ewald method (75) was applied to treat long-range electrostatic interactions, with a 1.2 nm real-space contribution cutoff for Coulombic interactions. A temperature of 310 K° and a pressure of 1 atm were maintained by the Langevin thermostat and barostat, respectively (76, 77). In all systems, the protonation states of peptides were assigned based on calculations at pH 7 and with 150 mM NaCl. First, a 1000-step minimization followed by 0.5 ns of equilibration with the protein constraint was performed to guide the system to the nearest local energy minimum in configuration space. After the equilibration process, all simulations were performed for 100 ns under an isothermal−isobaric ensemble without any restraints.

For each molecular dynamics, the RMSD and the BSA during the entire simulation were calculated. The former was obtained using the *RMSD Trajectory Tool* implemented in the software VMD 1.9.4a (78) (https://www.ks.uiuc.edu/Development/Download/download.cgi?PackageName=VMD) while the latter was calculated with an *in-house* developed code using the equation from Pandit *et al.*, 2020 (79). To estimate the relative binding free energy of each evaluated complex in the last 10 ns of simulation, the MM-GBSA (MM, molecular mechanics; GB, generalized Born; SA, surface area) method was used (80). The interaction analysis between residues from PT and α_1_AT was carried out using the Python (https://www.python.org) package ProLIF v.1.0.0 (81). Finally, the alanine scanning was performed for the last frame of the most stable complex using the server Mutabind2 (https://lilab.jysw.suda.edu.cn/research/mutabind2/) (36). For this, three different approaches were used, (i) mutate all residues simultaneously to alanine, (ii) cluster-based mutations: residues were separated into specific clusters and mutated each cluster, (iii) individual mutations.

### qPCR and mouse infection

C57BL/6 pairs (Jackson Laboratory) were bred in-house, and seven-day-old pups were treated in accordance with and approved by the University of Maryland, Baltimore (UMB), Institutional Animal Care and Use Committee (AUP 1122017). A streptomycin resistant derivative of *B. pertussis* Tohama I was grown on Bordet-Gengou (BG) agar supplemented with 200 μg/ml streptomycin at 37 °C for two days and resuspended to an *A*_600_ nm of 1.0 in PBS. Bacterial inoculum or vehicle PBS control was administered *via* a nebulizer system (Pari Vios) for 20 min. At 8 days post inoculation, mice were euthanized, and the left lung lobe and spleen were homogenized and plated on BG agar in serial dilutions to determine bacterial load. RNA was isolated from the superior lobe using TRIzol reagent (Invitrogen) and reverse transcribed using a High Capacity cDNA Reverse Transcription Kit (Applied Biosystems) as per the manufacturer's instructions. Quantitative real-time PCR was performed with PowerUp SYBR Green Master Mix (Applied Biosystems) in an Applied Biosystems 7500 Fast real-time PCR system. Transcript levels of serpinA1a-e, using primers 5‘-GCAGAAGGTTAGTCCAGATCC-3’ and 5‘-CTTCAGGGGAGCATTCTCC-3’, were normalized to the hypoxanthine phosphoribosyltransferase housekeeping gene, using primers 5′-GCTGACCTGCTGGATTACATTAA-3′ and 5′-GATCATTACAGTAGCTCTTCAGTCTG-3′, and compared with those from PBS-inoculated age-matched mice (calculated by 2^−ΔΔCT^).

### Treatment study

Human plasma-derived α_1_AT (Sigma-Aldrich, cat#A9042) was resuspended in PBS to 5 mg/ml, filter sterilized, aliquoted, and stored at −80 °C. C57BL/6 pups were inoculated as described in 4.15 and intraperitoneally injected with α_1_AT at 50 mg/kg or vehicle control using a 29G insulin syringe (Becton Dickinson). Treatment was repeated every two days starting immediately after aerosol inoculation on day 0. At 8 dpi, mice were euthanized, and the blood, lungs, and spleen were harvested to evaluate leukocytosis, bacterial load, and lung pathology. Leukocytosis was determined from blood acquired by cardiac puncture and treated with ammonium chloride-potassium lysis buffer. The left lung lobe and spleen were homogenized in PBS, and serial dilutions were plated on BG agar supplemented with streptomycin to quantify bacterial load. The inferior right lung lobe was fixed in 10% (wt/vol) formalin, mounted, and H&E stained by the UMB Pathology EM and Histology Laboratory.

### Reproducibility of experiments and statistics

All experiments were performed independently from each other at least three times. The number of replicates (n) for experiments or tested conditions is given in the figure legends. Representative results are shown in the figures. If not stated otherwise in the figure legends, the statistical analysis performed was a one-way ANOVA in combination with Dunnett’s multiple comparison test using GraphPad Prism Version 9 (GraphPad Software Inc., San Diego, CA, USA). The obtained *p* values are depicted as follows: ns = not significant *p* > 0.05, ∗*p* < 0.05, ∗∗*p* < 0.01, ∗∗∗*p* < 0.001, ∗∗∗∗*p* < 0.0001.

## Data availability

The datasets generated and/or analyzed during the current study are either included in the study or available from the corresponding author on reasonable request.

## Supporting information

This article contains supporting information.

## Ethics

Human HF was collected from multiple donors exhibiting the patient’s written informed consent prior to inclusion in the study and based on the positive vote 91/17 of the Ethics Commission of University of Ulm.

## Conflicts of interest

The authors declare that they have no conflicts of interest with the contents of this article.

## References

[1] Yeung K.H.T., Duclos P., Nelson E.A.S., Hutubessy R.C.W. (2017). An update of the global burden of pertussis in children younger than 5 years: a modelling study. Lancet Infect. Dis..

[2] Locht C., Antoine R. (2021). The history of pertussis toxin. Toxins (Basel).

[3] Bagcchi S. (2023). Pertussis cases rise in Denmark. Lancet Infect. Dis..

[4] Dalby T. (2024). Clarifying pertussis in Denmark. Lancet Infect. Dis..

[5] Mattoo S., Cherry J.D. (2005). Molecular pathogenesis, epidemiology, and clinical manifestations of respiratory infections due to Bordetella pertussis and other Bordetella subspecies. Clin. Microbiol. Rev..

[6] Scanlon K., Skerry C., Carbonetti N. (2019). Association of pertussis toxin with severe pertussis disease. Toxins (Basel).

[7] Carbonetti N.H. (2015). Contribution of pertussis toxin to the pathogenesis of pertussis disease. Pathog. Dis..

[8] Surridge J., Segedin E.R., Grant C.C. (2007). Pertussis requiring intensive care. Arch. Dis. Child.

[9] Connelly C.E., Sun Y., Carbonetti N.H. (2012). Pertussis toxin exacerbates and prolongs airway inflammatory responses during Bordetella pertussis infection. Infect. Immun..

[10] Hiramatsu Y., Suzuki K., Nishida T., Onoda N., Satoh T., Akira S. (2022). The mechanism of pertussis cough revealed by the mouse-coughing model. mBio.

[11] Stein P.E., Boodhoo A., Armstrong G.D., Cockle S.A., Klein M.H., Read R.J. (1994). The crystal structure of pertussis toxin. Structure.

[12] Tamura M., Nogimori K., Murai S., Yajima M., Ito K., Katada T. (1982). Subunit structure of islet-activating protein, pertussis toxin, in conformity with the A-B model. Biochemistry.

[13] Weiss A.A., Johnson F.D., Burns D.L. (1993). Molecular characterization of an operon required for pertussis toxin secretion. Proc. Natl. Acad. Sci. U. S. A..

[14] Burns D.L. (2021). Secretion of pertussis toxin from Bordetella pertussis. Toxins (Basel).

[15] Witvliet M.H., Burns D.L., Brennan M.J., Poolman J.T., Manclark C.R. (1989). Binding of pertussis toxin to eucaryotic cells and glycoproteins. Infect. Immun..

[16] Armstrong G.D., Howard L.A., Peppler M.S. (1988). Use of glycosyltransferases to restore pertussis toxin receptor activity to asialoagalactofetuin. J. Biol. Chem..

[17] Plaut R.D., Carbonetti N.H. (2008). Retrograde transport of pertussis toxin in the mammalian cell. Cell. Microbiol..

[18] el Bayâ A., Linnemann R., von Olleschik-Elbheim L., Robenek H., Schmidt M.A. (1997). Endocytosis and retrograde transport of pertussis toxin to the Golgi complex as a prerequisite for cellular intoxication. Eur. J. Cell Biol..

[19] Burns D.L., Manclark C.R. (1986). Adenine nucleotides promote dissociation of pertussis toxin subunits. J. Biol. Chem..

[20] Hazes B., Boodhoo A., Cockle S.A., Read R.J. (1996). Crystal structure of the pertussis toxin-ATP complex: a molecular sensor. J. Mol. Biol..

[21] Plaut R.D., Scanlon K.M., Taylor M., Teter K., Carbonetti N.H. (2016). Intracellular disassembly and activity of pertussis toxin require interaction with ATP. Pathog. Dis..

[22] Banerjee T., Cilenti L., Taylor M., Showman A., Tatulian S.A., Teter K. (2016). Thermal unfolding of the pertussis toxin S1 subunit facilitates toxin translocation to the cytosol by the mechanism of endoplasmic reticulum-associated degradation. Infect. Immun..

[23] Pande A.H., Moe D., Jamnadas M., Tatulian S.A., Teter K. (2006). The pertussis toxin S1 subunit is a thermally unstable protein susceptible to degradation by the 20S proteasome. Biochemistry.

[24] Worthington Z.E.V., Carbonetti N.H. (2007). Evading the proteasome: absence of lysine residues contributes to pertussis toxin activity by evasion of proteasome degradation. Infect. Immun..

[25] Ernst K., Mittler A.K., Winkelmann V., Kling C., Eberhardt N., Anastasia A. (2021). Pharmacological targeting of host chaperones protects from pertussis toxin in vitro and in vivo. Sci. Rep..

[26] Ernst K., Eberhardt N., Mittler A.K., Sonnabend M., Anastasia A., Freisinger S. (2018). Pharmacological cyclophilin inhibitors prevent intoxication of mammalian cells with Bordetella pertussis toxin. Toxins.

[27] Kellner A., Cherubin P., Harper J.K., Teter K. (2021). Proline isomerization as a key determinant for Hsp90-toxin interactions. Front. Cell Infect. Microbiol..

[28] Kellner A., Taylor M., Banerjee T., Britt C.B.T., Teter K. (2019). A binding motif for Hsp90 in the A chains of ADP-ribosylating toxins that move from the endoplasmic reticulum to the cytosol. Cell. Microbiol..

[29] Bokoch G.M., Katada T., Northup J.K., Hewlett E.L., Gilman A.G. (1983). Identification of the predominant substrate for ADP-ribosylation by islet activating protein. J. Biol. Chem..

[30] Katada T., Ui M. (1982). Direct modification of the membrane adenylate cyclase system by islet-activating protein due to ADP-ribosylation of a membrane protein. Proc. Natl. Acad. Sci. U. S. A..

[31] Bosso M., Ständker L., Kirchhoff F., Münch J. (2018). Exploiting the human peptidome for novel antimicrobial and anticancer agents. Bioorg. Med. Chem..

[32] Gadek J.E., Klein H.G., Holland P.V., Crystal R.G. (1981). Replacement therapy of alpha 1-antitrypsin deficiency. Reversal of protease-antiprotease imbalance within the alveolar structures of PiZ subjects. J. Clin. Invest..

[33] Schlömmer C., Brandtner A., Bachler M. (2021). Antithrombin and its role in host defense and inflammation. Int. J. Mol. Sci..

[34] Zhang Y., Zhang M., Tan L., Pan N., Zhang L. (2019). The clinical use of Fondaparinux: a synthetic heparin pentasaccharide. Prog. Mol. Biol. Transl Sci..

[35] Xue L.C., Rodrigues J.P., Kastritis P.L., Bonvin A.M., Vangone A. (2016). PRODIGY: a web server for predicting the binding affinity of protein–protein complexes. Bioinformatics.

[36] Zhang N., Chen Y., Lu H., Zhao F., Alvarez R.V., Goncearenco A. (2020). MutaBind2: predicting the impacts of single and multiple mutations on protein-protein interactions. iScience.

[37] Barthold L., Heber S., Schmidt C.Q., Gradl M., Weidinger G., Barth H., Fischer S. (2022). Human α-defensin-6 neutralizes Clostridioides difficile toxins TcdA and TcdB by direct binding. Int. J. Mol. Sci..

[38] Paddock C.D., Sanden G.N., Cherry J.D., Gal A.A., Langston C., Tatti K.M. (2008). Pathology and pathogenesis of fatal Bordetella pertussis infection in infants. Clin. Infect. Dis..

[39] Liu C., Yang L., Cheng Y., Xu H., Xu F. (2020). Risk factors associated with death in infants <120 days old with severe pertussis: a case-control study. BMC Infect. Dis..

[40] Winter K., Zipprich J., Harriman K., Murray E.L., Gornbein J., Hammer S.J. (2015). Risk factors associated with infant deaths from pertussis: a case-control study. Clin. Infect. Dis..

[41] Scanlon K.M., Snyder Y.G., Skerry C., Carbonetti N.H. (2017). Fatal pertussis in the neonatal mouse model is associated with pertussis toxin-mediated pathology beyond the airways. Infect. Immun..

[42] Scanlon K.M., Chen L., Carbonetti N.H. (2022). Pertussis toxin promotes pulmonary hypertension in an infant mouse model of Bordetella pertussis infection. J. Infect. Dis..

[43] Dasí F. (2023). Alpha-1 antitrypsin deficiency. Med. Clin. (Barc).

[44] Paterson T., Moore S. (1996). The expression and characterization of five recombinant murine alpha 1-protease inhibitor proteins. Biochem. Biophys. Res. Commun..

[45] Sugimoto M., Nakanishi Y., Otokawa M., Uchida N., Yasuda T., Sato H., Sato Y. (1983). Effect of Bordetella pertussis leukocytosis (lymphocytosis)-promoting factor (LPF) on the physical lymphoepithelial-cell association studied with the use of an in vitro model of mouse thymus. J. Immunol..

[46] Travis J., Salvesen G.S. (1983). Human plasma proteinase inhibitors. Annu. Rev. Biochem..

[47] Janciauskiene S., Welte T. (2016). Well-known and less well-known functions of alpha-1 antitrypsin. Its role in chronic obstructive pulmonary disease and other disease developments. Ann. Am. Thorac. Soc..

[48] Wewers M.D., Casolaro M.A., Crystal R.G. (1987). Comparison of alpha-1-antitrypsin levels and antineutrophil elastase capacity of blood and lung in a patient with the alpha-1-antitrypsin phenotype null-null before and during alpha-1-antitrypsin augmentation therapy. Am. Rev. Respir. Dis..

[49] Crystal R.G. (1990). Alpha 1-antitrypsin deficiency, emphysema, and liver disease. Genetic basis and strategies for therapy. J. Clin. Invest..

[50] Hubbard R.C., Crystal R.G. (1988). Alpha-1-antitrypsin augmentation therapy for alpha-1-antitrypsin deficiency. Am. J. Med..

[51] Hubbard R.C., Sellers S., Czerski D., Stephens L., Crystal R.G. (1988). Biochemical efficacy and safety of monthly augmentation therapy for alpha 1-antitrypsin deficiency. JAMA.

[52] Campos M.A., Kueppers F., Stocks J.M., Strange C., Chen J., Griffin R. (2013). Safety and pharmacokinetics of 120 mg/kg versus 60 mg/kg weekly intravenous infusions of alpha-1 proteinase inhibitor in alpha-1 antitrypsin deficiency: a multicenter, randomized, double-blind, crossover study (SPARK). COPD.

[53] Karadagi A., Johansson H., Zemack H., Salipalli S., Mörk L.M., Kannisto K. (2017). Exogenous alpha 1-antitrypsin down-regulates SERPINA1 expression. PLoS One.

[54] Wettstein L., Weil T., Conzelmann C., Müller J.A., Groß R., Hirschenberger M. (2021). Alpha-1 antitrypsin inhibits TMPRSS2 protease activity and SARS-CoV-2 infection. Nat. Commun..

[55] Long G.L., Chandra T., Woo S.L.C., Davie E.W., Kurachi K. (1984). Complete sequence of the cDNA for human .alpha.1-antitrypsin and the gene for the S variant. Biochemistry.

[56] Lietz S., Sokolowski L.-M., Barth H., Ernst K. (2024). Alpha-1 antitrypsin inhibits Clostridium botulinum C2 toxin, Corynebacterium diphtheriae diphtheria toxin and B. anthracis fusion toxin. Sci. Rep..

[57] Hubbard R.C., McElvaney N.G., Sellers S.E., Healy J.T., Czerski D.B., Crystal R.G. (1989). Recombinant DNA-produced alpha 1-antitrypsin administered by aerosol augments lower respiratory tract antineutrophil elastase defenses in individuals with alpha 1-antitrypsin deficiency. J. Clin. Invest..

[58] Pinto M.V., Merkel T.J. (2017). Pertussis disease and transmission and host responses: insights from the baboon model of pertussis. J. Infect..

[59] Rodríguez-Alfonso A., Heck A., Ruiz-Blanco Y.B., Gilg A., Ständker L., Kuan S.L. (2022). Advanced EPI-X4 derivatives covalently bind human serum albumin resulting in prolonged plasma stability. Int. J. Mol. Sci..

[60] Zhang J., Xin L., Shan B., Chen W., Xie M., Yuen D. (2012). PEAKS DB: de novo sequencing assisted database search for sensitive and accurate peptide identification. Mol. Cell Proteomics.

[61] von Eichel-Streiber C., Harperath U., Bosse D., Hadding U. (1987). Purification of two high molecular weight toxins of Clostridium difficile which are antigenically related. Microb. Pathog..

[62] Ashok Y., Miettinen M., Oliveira D.K.H.d., Tamirat M.Z., Näreoja K., Tiwari A. (2020). Discovery of compounds inhibiting the ADP-ribosyltransferase activity of pertussis toxin. ACS Infect. Dis..

[63] Osseforth C., Moffitt J.R., Schermelleh L., Michaelis J. (2014). Simultaneous dual-color 3D STED microscopy. Opt. Express, OE.

[64] Weng G., Wang E., Wang Z., Liu H., Zhu F., Li D., Hou T. (2019). HawkDock: a web server to predict and analyze the protein-protein complex based on computational docking and MM/GBSA. Nucleic Acids Res..

[65] Zacharias M. (2003). Protein-protein docking with a reduced protein model accounting for side-chain flexibility. Protein Sci..

[66] Feng T., Chen F., Kang Y., Sun H., Liu H., Li D. (2017). HawkRank: a new scoring function for protein–protein docking based on weighted energy terms. J. Cheminformatics.

[67] Hou T., Qiao X., Zhang W., Xu X. (2002). Empirical aqueous solvation models based on accessible surface areas with implicit electrostatics. J. Phys. Chem. B.

[68] Tovchigrechko A., Vakser I.A. (2006). GRAMM-X public web server for protein–protein docking. Nucleic Acids Res..

[69] Abramson J., Adler J., Dunger J., Evans R., Green T., Pritzel A. (2024). Accurate structure prediction of biomolecular interactions with AlphaFold 3. Nature.

[70] Phillips J.C., Braun R., Wang W., Gumbart J., Tajkhorshid E., Villa E. (2005). Scalable molecular dynamics with NAMD. J. Comput. Chem..

[71] Vanommeslaeghe K., Hatcher E., Acharya C., Kundu S., Zhong S., Shim J. (2010). CHARMM general force field: a force field for drug-like molecules compatible with the CHARMM all-atom additive biological force fields. J. Comput. Chem..

[72] Best R.B., Zhu X., Shim J., Lopes P.E.M., Mittal J., Feig M., Mackerell A.D. (2012). Optimization of the additive CHARMM all-atom protein force field targeting improved sampling of the backbone ϕ, ψ and side-chain χ1 and χ2 dihedral angles. J. Chem. Theor. Comput..

[73] Jorgensen W.L., Chandrasekhar J., Madura J.D., Impey R.W., Klein M.L. (1983). Comparison of simple potential functions for simulating liquid water. J. Chem. Phys..

[74] Cuendet M.A., van Gunsteren W.F. (2007). On the calculation of velocity-dependent properties in molecular dynamics simulations using the leapfrog integration algorithm. J. Chem. Phys..

[75] Darden T., York D., Pedersen L. (1993). Particle mesh Ewald: an N⋅log(N) method for Ewald sums in large systems. J. Chem. Phys..

[76] Davidchack R.L., Handel R., Tretyakov M.V. (2009). Langevin thermostat for rigid body dynamics. J. Chem. Phys..

[77] Feller S.E., Zhang Y., Pastor R.W., Brooks B.R. (1995). Constant pressure molecular dynamics simulation: the Langevin piston method. J. Chem. Phys..

[78] Humphrey W., Dalke A., Schulten K. (1996). VMD: visual molecular dynamics. J. Mol. Graphics.

[79] Pandit G., Biswas K., Ghosh S., Debnath S., Bidkar A.P., Satpati P. (2020). Rationally designed antimicrobial peptides: insight into the mechanism of eleven residue peptides against microbial infections. Biochim. Biophys. Acta Biomembr..

[80] Wang E., Sun H., Wang J., Wang Z., Liu H., Zhang J.Z.H., Hou T. (2019). End-point binding free energy calculation with MM/PBSA and MM/GBSA: strategies and applications in drug design. Chem. Rev..

[81] Bouysset C., Fiorucci S. (2021). ProLIF: a library to encode molecular interactions as fingerprints. J. Cheminformatics.

