# Supplementary material for: Alpha-1 antitrypsin inhibits pertussis toxin
Source: J Biol Chem. 2024 Oct 30;300(12):107950. doi: 10.1016/j.jbc.2024.107950 (PMC11683240; doi:10.1016/j.jbc.2024.107950)
Supplement: Supporting information [file mmc1.docx]

**Supplementary Figures - Alpha1-antitrypsin inhibits pertussis toxin**

**Supplementary Figure 1. General strategy for obtaining the peptide library and further purification of the biologically active compound.** A sample of 1000 L HF was ultrafiltered, and the fraction containing peptides and proteins of MW < 30 kDa was loaded onto a cation-exchanger (Sulfopropyl) and subjected to stepwise elution by increasing pH, generating pools E1 to E4. The non-retained fraction was loaded onto Amberlite XAD and subjected to stepwise elution by increasing the concentration of acetonitrile to generate pools E5 to E8. Pools E1 to E8 were subjected separately to reversed-phase chromatography (Sepax Poly RP-300). Every pool generated 55 fractions, so the peptide library was composed of 440 fractions in total. Small aliquots from the peptide bank were biologically evaluated, and the active fractions (34, 35, 36, and 41 from pool E4) were subjected to several purification rounds guided by biological evaluation until the isolation of a high-purity chromatographic fraction that was analyzed by mass spectrometry, being alpha-1 antitrypsin identified as the dominant molecule in the sample.

**Supplementary Figure 2. α_1_AT was identified from a hemofiltrate library as a PT inhibitor.** **(a-d)** Chromatographic profiles of active fractions 34, 35, 36, and 41 in RP-HPLC. Derived 93 fractions were analyzed for activity against PT intoxication, revealing subfractions 34_53-56, 35_48-49, and 36_59-60 as the most promising ones. **(e-l)** Chromatographic profiles of 34_53-56, 35_48-49, and 36_59-60, resulting in 408 screened fractions, and biological evaluation showing 34_55_56 to 58 as the most active ones. **(a-l)** PT (10 ng/ml) and 20 µl of subfraction / subsubfractions or the respective amount of solvent (H_2_O) were added directly to CHO-K1 cells in FCS-free medium and incubated for 4 h at 37 °C. Cells were left untreated as further control (Con). After the incubation, the cells were lysed and Gαi, which had not been ADP-ribosylated during the intoxication with PT, was ADP-ribosylated and biotin-labeled via the incubation with PTS1 and biotin-labeled NAD^+^. Subsequently, the biotin-labeled Gαi was detected via Western Blot, while Hsp90 or Ponceau-S staining served as control for equal protein loading. The intensity values of the bar graph are given as x-fold of the untreated control (Con), normalized to Ponceau-S staining, mean +/- SD (at least n = 1 at most n = 2 from two independent experiments). (Blue line: chromatogram from chromatographic fractionation process, grey bars = screening result, green bars: screening results that were considered as hits and or subjected to further chromatographic fractionation). Abbreviations are used to identify all chromatographic fractions. From left to right, the number of the active chromatographic fraction used for each next fractionation step is given in chronological order. For example, 34_55_56 to 58 is referred to as the chromatographic fractions 56 to 58 derived from fraction 34_55, obtained from fraction 34.

**Supplementary Figure 3. Effect of α_1_AT on PT-intoxication of CHO-K1 cells.** **(a-b)** PT (10 ng/ml) and α_1_AT or the respective amount of solvent (H_2_O) were added directly to CHO-K1 cells in FCS-free medium and incubated for 4 h at 37 °C. Cells were left untreated as further control (Con) or incubated with α_1_AT only. After the incubation, the cells were lysed and Gαi, which had not been ADP-ribosylated during the intoxication with PT, was ADP-ribosylated and biotin-labeled via the incubation with PTS1 and biotin-labeled NAD^+^. Subsequently, the biotin-labeled Gαi was detected via Western Blot, while Hsp90 or Ponceau-S staining served as control for equal protein loading. The bar graph **(a)** shows the quantification of Western Blot signals from three independent experiments, while **(b)** shows results of a representative experiment. The intensity values of the bar graph are given as x-fold of the untreated control (Con), normalized to Hsp90 or Ponceau-S staining, mean +/- SEM (n = 9 values from three independent experiments). **(a)** Significance was tested using one-way ANOVA followed by Dunnett’s multiple comparison test and refers to untreated controls (con) (* p 0.1, ** p 0.01, *** p 0.001, **** p 0.0001, ns not significant).

**Supplementary Figure 4. Effect of α_1_AT on enzyme activity of PTS1 *in vitro*.** **(a**) Schematic representation of experimental setup for the enzyme activity assay using recombinant PTS1 and Gαi. PTS1 and α_1_AT were pre-incubated for 15 min before addition of Gαi and biotin-NAD^+^ and the incubation for 40 min at room temperature. **(b-c)** PTS1 (84 nM) and different concentrations α_1_AT or the respective amount of solvent (H_2_O) (con) were pre-incubated for 15 min at room temperature. Then Gαi (825 nM) and biotin-NAD^+^ were added and incubated for 40 min at room temperature. Gαi, which was ADP-ribosylated and biotin-labeled via the incubation with PTS1 and biotin-labeled NAD^+^, was detected via Western Blot. The bar graph **(b)** shows the quantifications of Western Blot signals from ten independent experiments, while **(c, d, e)** show blots of a representative experiments. The intensity values of the bar graph are given as x-fold of the control (con), mean +/- SEM (at least n = 6 at most n = 20 from ten independent experiments). **(b)** Significance was tested using one-way ANOVA followed by Dunnett’s multiple comparison test and refers to untreated controls (con) (* p 0.1, ** p 0.01, *** p 0.001, **** p 0.0001, ns not significant).

**Supplementary Figure 5. Effect of α_1_AT on detectable PST1 signal in CHO-K1 and A549 cells.** **(a)** PT (50 ng/ml) and different concentrations α_1_AT or the respective amount of solvent (H_2_O) were added directly to CHO-K1 cells and incubated for 4 h at 37 °C. Cells were left untreated as control (Con). Subsequently, the cells were washed, fixed, permeabilized (as indicated), and quenching was performed. Blocking was performed, and the cells were incubated with primary antibodies for PTS1 (green) and α_1_AT (red). Primary antibodies were detected via fluorescently labeled secondary antibodies and nuclei were stained using Hoechst (blue). Representative images are shown from three independent experiments (n = 3). **(b)** PT (50 ng/ml) and α_1_AT or the respective amount of solvent (H_2_O) were added directly to A549 cells and incubated for 4 h at 37 °C. Subsequently, the experiment was performed as described in **(a)**. Representative images are shown from three independent experiments (n = 3). Images of untreated controls are identical to control images of Figure 7.

**Supplementary Figure 6. Effect of α_1_AT on binding of PT in CHO-K1 cells.** PT (1 µg/ml) and different concentrations α_1_AT or the respective amount of solvent (H_2_O) were added directly to CHO-K1 cells and incubated for 40 min at 4 °C to enable PT binding but not internalization. Cells were left untreated as control. Subsequently, the cells were washed, fixed, permeabilized (as indicated), and quenching was performed. Blocking was performed, and the cells were incubated with primary antibodies for PTS1 (green) and α_1_AT (red). Primary antibodies were detected via fluorescently labeled secondary antibodies, and nuclei were stained using Hoechst (blue). Representative images are shown from three independent experiments (n = 3).

**Supplementary Figure 7. Effect of α_1_AT on detectable PST1 signal in CHO-K1 cells analyzed by high-resolution stimulated emission depletion (STED) microscopy.** PT (50 ng/ml) and different concentrations α_1_AT or the respective amount of solvent (H_2_O) were added directly to CHO-K1 cells and incubated for 4 h at 37 °C. Cells were left untreated as control (Con). Subsequently, the cells were washed, fixed, permeabilized (as indicated), and quenching was performed. Blocking was performed and the cells were incubated with primary antibodies for PTS1 (green) and α_1_AT (red). Primary antibodies were detected via respective secondary antibodies. Representative images are shown from three independent experiments (n = 2-3). The scale bar represents 10 µm.

**

Supplementary Figure 8. Selection of residues for mutagenesis study.** α_1_AT is represented in gray carton and pertussis toxin in colored surfaces, S1, green; S2, turquoise; S3, purple; S4, red; S5, yellow. Selected residues for mutagenesis are represented in spheres.

**Supplementary Table 1. *In silico* alanine scanning with the Mutabind2 server for residues in the interaction interface of the most stable α_1_AT-PT complex.** The evaluated residues correspond to those presenting more than 50 % of occupancy for any interaction. Highlighted in black are those ∆∆G values higher than the threshold (1.5 kcal/mol).

| **Residue** | **Approach 1** | | **Approach 2** | | | **Approach 3** |
| --- | --- | --- | --- | --- | --- | --- |
|  | **Cluster ID** | **∆∆G** | **Cluster ID** | **∆∆G** | **∆∆G** | **∆∆G** |
| E75A | M1 | **4.51** | 1 | **3.82** | 1.25 | 1.12 |
| E78A |  |  |  |  |  | 0.83 |
| T309A |  |  |  |  |  | 0.98 |
| K310A |  |  |  |  |  | 0.92 |
| S313A |  |  |  |  |  | 0.26 |
| N314A |  |  | 1/2 |  | **3.9** | **1.6** |
| K174A |  |  | 2 | **3.63** |  | 1.36 |
| E175A |  |  |  |  |  | 1.48 |
| L176A |  |  |  |  |  | 0.91 |
| D177A |  |  |  |  |  | 1.46 |
| R178A |  |  |  |  |  | 0.69 |
| D179A |  |  |  |  |  | 1.39 |
| Q44A |  |  | 3 | **2.77** | **2.71** | 0.5 |
| S45A |  |  |  |  |  | 0.89 |
| S301A |  |  |  |  |  | 0.46 |
| Q305A |  |  |  |  |  | 0.85 |

**Supplementary Figure 9. Interaction and aggregate formation of α_1_AT with PT and PTS1 *in vitro*. (a)** Decreasing concentrations of PTS1 (antibody control), Gαi, Gαi w/o His-tag, and α_1_AT were vacuum aspirated onto a nitrocellulose membrane using the Dot bot system. PBS was aspirated as a control. Subsequently, the membrane was stained with Ponceau-S (right), blocked, and cut for incubation with the overlays, PT, PTS1, and PBS-T as control. Bound PTS1 was detected using an antibody against PTS1 (left). A representative blot is shown for at least three independent experiments (n = 3). **(b)** PT (100 ng) and α_1_AT (100 µM) or the respective amount of solvent (H_2_O), as wells as TcdB (50 ng) and α-Defensin-6 (6 µM) (positive control for precipitation), α_1_AT (100 µM) or the respective amount of solvent (H_2_O) were incubated for 30 min. Supernatant and pellet were collected after centrifugation of the samples and proceeded to gel electrophoresis and Western blotting. PT was detected using an anti-PTS1 antibody and TcdB was detected using an anti-TcdB antibody. A representative blot is shown for at least three independent experiments (n = 3) for PT and two independent experiments for TcdB (n = 2).


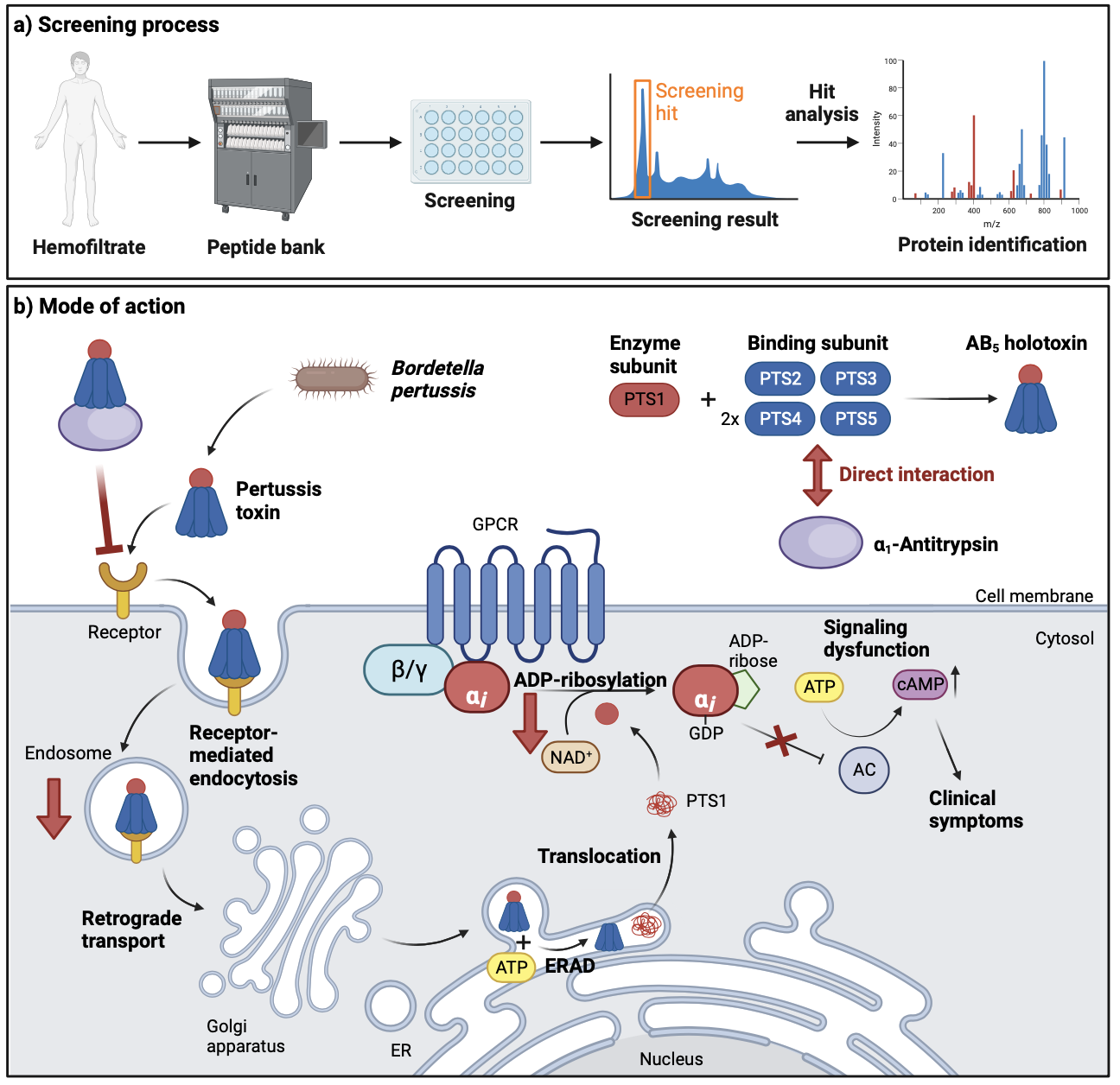


**Supplementary Figure 10. Overview on the screening process and mode of action of PT and α_1_AT.** **(a)** Overview of the screening process, which revealed α_1_AT as an inhibitor of PT. **(b)** Overview of the mode of action of PT and α_1_AT. α_1_AT inhibits intoxication of cells via the inhibition of binding of PT to cells through direct interaction with the binding subunit of the toxin. Created in BioRender. Lietz, S. (2022) https://BioRender.com/x61a690
